# Disentangling the Calorimetric Glass-Transition Trace in Polymer/Oligomer Mixtures from the Modeling of Dielectric Relaxation and the Input of Small-Angle Neutron Scattering

**DOI:** 10.1021/acs.macromol.2c00609

**Published:** 2022-08-22

**Authors:** Numera Shafqat, Angel Alegría, Arantxa Arbe, Nicolas Malicki, Séverin Dronet, Lionel Porcar, Juan Colmenero

**Affiliations:** 1 Materials Physics Center (MPC), Centro de Física de Materiales (CSIC, UPV/EHU), Paseo Manuel de Lardizabal 5, 20018 San Sebastián, Spain; 2 Manufacture Française des Pneumatiques MICHELIN, Site de Ladoux, 23 Place des Carmes Déchaux, Cedex 63040 Clermont-Ferrand, France; 3 Departamento de Polímeros y Materiales Avanzados: Física, Química y Tecnología (UPV/EHU), Facultad de Química, Universidad del Pais Vasco, 20018 San Sebastián, Spain; 4 Institut Laue-Langevin, 71 Avenue des Martyrs, Grenoble Cedex 9, 38042, France; 5 Donostia International Physics Center, Paseo Manuel de Lardizabal 4, 20018 San Sebastián, Spain

## Abstract

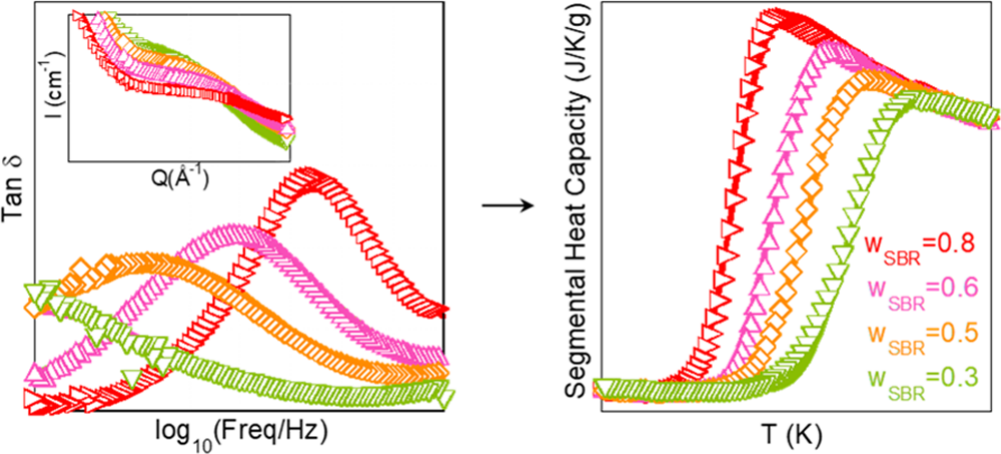

We have disentangled the contributions to the glass transition as observed by differential scanning calorimetry (DSC) on simplified systems of industrial interest consisting of blends of styrene–butadiene rubber (SBR) and polystyrene (PS) oligomer. To do this, we have started from a model previously proposed to describe the effects of blending on the equilibrium dynamics of the α-relaxation as monitored by broadband dielectric spectroscopy (BDS). This model is based on the combination of self-concentration and thermally driven concentration fluctuations (TCFs). Considering the direct insight of small-angle neutron scattering on TCFs, blending effects on the α-relaxation can be fully accounted for by using only three free parameters: the self-concentration of the components φ_self_^SBR^ and φ_self_^PS^) and the relevant length scale of segmental relaxation, 2*R*_c_. Their values were determined from the analysis of the BDS results on these samples, being that obtained for 2*R*_c_ ≈ 25Å in the range usually reported for this magnitude in glass-forming systems. Using a similar approach, the distinct contributions to the DSC experiments were evaluated by imposing the dynamical information deduced from BDS and connecting the component segmental dynamics in the blend above the glass-transition temperature *T*_g_ (at equilibrium) and the way the equilibrium is lost when cooling toward the glassy state. This connection was made through the α-relaxation characteristic time of each component at *T*_g_, τ_g_. The agreement of such constructed curves with the experimental DSC results is excellent just assuming that τ_g_ is not affected by blending.

## 1 Introduction

Differential scanning calorimetry (DSC) is probably the widest used technique to corroborate miscibility in polymer blends, since the most extended traditional criterion for miscibility is the observation of a single glass-transition temperature *T*_g_ in the calorimetric trace.^1−3^ Contrarily to the rather abrupt step in the specific heat *C*_*p*_ of homopolymers, blends usually show a monotonic increase in *C*_*p*_ extending over a broad temperature range between the two *T*_g_s of the neat polymers.^4,5^ The position and broadening of these extended steps depend on composition. As for the homopolymers, from the inflection point of *C*_*p*_, a glass-transition temperature can be deduced for the blend. Traditionally, it was believed that miscibility implies a single glass transition for the blend components.^1,2^ This concept was, however, critically revised in light of extensive investigations on polymer blend dynamics by means of different methods including, e.g., broadband dielectric spectroscopy (BDS), nuclear magnetic resonance (NMR), and quasielastic neutron scattering (QENS).^5−13^ These techniques have the advantage that they can be sensitive to a given component (if it has a much stronger dipole moment than the other, in the case of BDS, or applying selective deuterium labeling, in the case of QENS). In addition, they address dynamical processes in equilibrium—the dipolar reorientations or the atomic motions in the α-relaxation—instead of following a loss of thermodynamic equilibrium, as it is the case of DSC experiments. Intuitively, for a perfect and homogeneous blend one would expect to observe only a single average relaxation time for the α-relaxation. This would translate into a single *T*_g_ measured by DSC. On the contrary, even in thermodynamically miscible blends two different mean relaxation times are usually found, each of them corresponding to the dynamics of the α-relaxation of each component modified by blending.^5^ This finding is known as dynamic heterogeneity of miscible blends. Dynamic heterogeneity is particularly prominent in so-called dynamically asymmetric blends (mixtures where the *T*_g_s of the neat components display a large difference, like, for example, poly(methyl methacrylate) (PMMA) and poly(ethylene oxide) (PEO)), and its establishment broke the paradigm of the presence of a single *T*_g_ in miscible polymer blends. In fact, two different calorimetric *T*_g_s have been resolved in some dynamically asymmetric polymer blends including the showcase of PMMA/PEO.^3,4,8,14^ These results can be considered as additional evidence of the close connection of the slowing down of the α-relaxation dynamics and the loss of the thermodynamic equilibrium associated with the glass formation process. As a general rule, it is considered that for polymers and other glass-forming systems the time scale of the α-relaxation at the glass transition temperature, as determined at usual heating/cooling rates of some K/min, is in the range of 10–100 s.^15^

The origin of the dynamic heterogeneity in blends is nowadays attributed to self-concentration (SC) effects:^16−18^ the local concentration around one segment of one of the blend components is always richer in this component due to the chain connectivity. Since the average *T*_g_ in the blend depends on composition, both components experience different “effective glass transitions” *T*_g,eff_s. This implicitly translates into different relaxation times for both components, i.e., the observation of dynamic heterogeneity in the system. Dynamic heterogeneity is however not the only effect of blending on the dynamic properties of miscible blends. The other main general observation is the broadening of the relaxation function as, for example, monitored by BDS. This effect is believed to be due to the thermally driven concentration fluctuations (TCFs), which are always present in a two-component system in equilibrium.^5,6,8,11,19,20^

To describe the DSC results on blends, usually quasi-phenomenological mixing rules such as the Fox,^21^ diMarzio,^22^ or the Kwei^23,24^ equations have been proposed in order to reproduce the concentration dependence of the average value of the glass-transition temperature. Reference (25) provides an interesting updated discussion on the grounds of the different approaches found in the literature. In a further step, taking into account the SC concept and applying the Fox equation to deduce the *T*_g,eff_s of the components, in refs (16 and 26) the concentration dependence of the average *T*_g_ of the blends of different pairs of polymers was reproduced and the shape of the DSC trace was qualitatively accounted for.^16^ Self-concentration effects were also considered in the attempt to reproduce the bimodal feature of the DSC trace of blends containing PEO in ref (27). Information about underlying distributions of glass-transition temperatures in the DSC results of blends was also extracted applying different methods in ref (28). However, to our knowledge, to date, the whole functional form of the DSC trace of polymer blends has not been quantitatively accounted for by any model or theoretical approach.

A full description of the DSC results implies describing not only the location of the midpoint or the inflection point(s) but also the broadening and shape of the trace, i.e., the details of how the contribution of the components to the whole curve behaves. It is worth noting that the DSC trace in the glass transition reflects the way thermodynamic equilibrium is lost when the α-relaxation time reaches laboratory time scales. Therefore, it is expected that DSC results should reflect in some manner both effects identified for the α-relaxation in equilibrium, dynamic asymmetry and broadening, that originate from SC and TCFs, respectively.

During the last years we have been investigating mixtures of interest in the tire industry, namely, blends of styrene–butadiene rubber (SBR) and polystyrene (PS) oligomers.^29−31^ The low molecular weight of PS facilitates miscibility with SBR. We found that SC and TCFs are also the main ingredients to determine blending effects in these simplified industrial systems. We proposed a model^29,30^ that combines these two factors to explain the BDS results of the blend at equilibrium, i.e., at *T > T*_g_. That model also explains the mechanical response at *T > T*_g_ of the same blends.^30^ The question we want to address now is whether—and how—the same framework can be used for determining the DSC traces in the *T*_g_ range of these mixtures.

In the application of the above-mentioned model, a series of parameters are involved to account for SC and TCF effects. In this context, small-angle neutron scattering (SANS) experiments on mixtures with enough scattering contrast are of utmost interest since they provide direct insight on the TCFs.^32−35^ This SANS information can be exploited to directly determine the impact of TCFs on the broadening of the α-relaxation and reduce the number of free parameters from the proposed model. In addition, SANS results also allow discerning the temperature/composition regions where the mixtures are thermodynamically miscible.^32−35^

With these ideas in mind, in this work we have combined DSC, BDS, and SANS experiments on simplified blends of industrial interest, composed again by SBR and PS oligomers. To provide SANS contrast, PS was deuterated and SBR protonated. All experiments here reported were carried out on the same samples (note that PS here is isotopically labeled and SBR has a different microstructure and molecular weight than in previous works^29−31^). Our final goal was to establish the underlying contributions to the DSC response for a wide range of compositions. To this end we connected the modeling of the segmental dynamics with the calorimetric behavior using the information deduced from the analysis of the BDS results supported by the SANS direct insight on TCFs. In this analysis, the determination of the relevant length scale for the α-relaxation is involved, a fundamental question recurrently emerging in the field of glass-forming systems.^36,37^ With our strategy, this length scale becomes the main parameter we need to fix in order to reproduce the blending broadening effect on the BDS relaxation function, for all temperatures and concentrations.

The paper is structured according to the strategy followed. We first present the bases of the model previously proposed to describe the effects of blending on the equilibrium α-relaxation, in particular as it is observed by BDS, as theoretical background. Thereafter the experimental details are given, regarding the samples investigated and the techniques included in the adopted methodology. In the Results section the experimentally obtained DSC traces are first presented, and the results corresponding to the neat components are modeled as a prerequisite for the later description of the blends results. Next, the SANS results revealing TCF are analyzed, with a twofold goal: (i) to determine the phase diagram and (ii) to provide the mean square of the TCF as a function of the length scale and blend composition. This information is imposed when applying the model to the BDS results, such that blending effects are accounted for by only three parameters: the self-concentration of both blend components and the relevant length scale of the α-relaxation. The values of these three parameters are determined from the BDS analysis. Thereafter, the connection of the model for the segmental dynamics of the mixtures with the calorimetric results is presented, and, making use of the previously gathered information, the DSC traces are computed and directly compared with the experimental results. The consistency of the proposed approach and implications of the assumptions made in the extension to describe the DSC traces are discussed before we summarize the conclusions of this work.

## 2 Background: Modeling the α-Relaxation of SBR/PS Blends

The model used previously for describing the α-relaxation of SBR/PS blends^29,30^ is based on thermally driven concentration fluctuations and self-concentration concepts. Following previous works, it is assumed that the TCFs evolve on a much longer time scale than that of the segmental relaxation. This entails that the polymer blend can be viewed as a set of subvolumes “*i*” each with a different SBR concentration, 0 ≤ φ_*i*_ ≤ 1. This quasi-static distribution of concentration *g*(φ_*i*_) in the blends can be described by a Gaussian function centered around the bulk concentration of the blend φ:
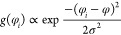
1where σ is the standard deviation of the distribution of concentration. When applying the model to describe the dielectric response of SBR and PS blends, the contributions of the components to the total dielectric permittivity of the blend can be written as
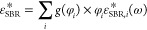
2a
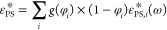
2bwhere ε_SBR,i_^*^(ω) and ε_PS,i_^*^(ω)—the complex dielectric permittivity associated with SBR and PS, respectively, in region “*i*”—are assumed to have the same characteristics of the relaxation of the corresponding homopolymers except the time scale. The time scale of the dynamics of a given polymer segment located in region “*i*” of a miscible blend is controlled by the local composition in a small region around the segment *c* of this component. This local composition is described by an effective concentration φ_eff,*i*_, which for the SBR and PS components is given by
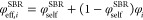
3a
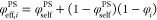
3b

We have introduced the self-concentration parameters, φ_self_^SBR^ and φ_self_^PS^, which will be assumed to be concentration independent. This is a crude approximation when using the self-concentration parameters for data fitting; however, it can be justified by considering their fundamental significance.^25^ Note that self-concentration was introduced in connection with both the relatively small size of the region around a given segment determining its dynamical behavior and the molecular characteristics (persistence length) of the particular component of the mixture.^16^

The relaxation time values of each component in a given subvolume are then calculated using the Vogel–Fulcher–Tammann (VFT) equation:^38−40^
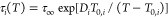
4

The same value of the prefactor τ_*∞*_ = 10^–13^ s (a typical vibrational frequency) is assumed for the pure components and for each component in any of the regions. The other VFT parameters, *D* (related with the so-called dynamic fragility) and *T*_0_ (Vogel temperature), are evaluated for the neat components *D*^SBR^, *T*_0_^SBR^, *D*^PS^, and *T*_0_^PS^ and obtained for each component in a given region “*i*” by using mixing rules with the corresponding effective concentrations. Particularly, a linear mixing rule is assumed for *D*_*i*_:
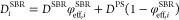
5a
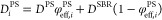
5b

For *T*_0,*i*_ we have used a Fox-like equation,^21^ following previous works;^30^ that is, *T*_0,*i*_ values are calculated as
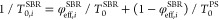
6a
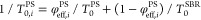
6b

In the framework of this model the total dielectric response of the blends is obtained summing up the contribution of each component in the blend.
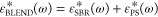
7

## 3 Experimental Section

### 3.1 Samples

Protonated styrene–butadiene rubber was synthesized by anionic polymerization by the Michelin Company. Before their use for copolymerization, the monomers were first dried over BuLi for butadiene and over calcium hydride and dibutylmagnesium for styrene and then distilled to obtain purified monomers. The copolymerization was initiated by butyllithium in methylcyclohexane at 50 °C. The deuterated polystyrene was purchased from Polymer Source, synthesized by living anionic polymerization of styrene-*d*_8_. Table 1 shows the microstructure, the average molecular weight (*M̅*_n_), and polydispersity (*M̅*_w_/*M̅*_n_) of the neat polymers.

**Table 1:** Molecular Weights, Polydispersities, and Weight Fractions of Styrene (S), 1,2-Butadiene (1,2-B), and 1,4-Butadiene (1,4-B) of the Pure Component Investigated.

sample	*w*_S_	*w*_1,2-B_	*w*_1,4-B_	*M̅*_n_ (kg/mol)	*M̅*_w_ (kg/mol)	PDI	*D* (g/cm^3^)
SBR	0.278	0.178	0.544	10.16	10.59	1.04	1.01
PS	0.94^a^			0.90	0.98	1.09	1.12

a Taking into account the weight of end-groups.

Taking into consideration the microstructure of SBR, for the analysis of the neutron scattering data we have defined an “effective monomer” composed by 0.167 styrene monomer and 0.833 butadiene monomer. Considering the density of the polymer, this effective monomer has a volume of SBR (*v*_SBR_) of 1.022 10^–22^ cm^3^, while that of PS (*v*_PS_) is 1.661 × 10^–22^ cm^3^. The scattering length density ρ (scattering length of the monomer divided by the monomeric volume) of SBR was calculated to be ρ_SBR_ = 8.50 × 10^9^ cm^–2^. In the case of PS, which is deuterated but not 100%, the scattering length density was experimentally determined in a previous work^31^ (ρ_PS_ = 59.25 × 10^9^ cm^–2^). We note that the values of some parameters as the effective monomer volume or the scattering length density are specific for the particular materials here investigated, since they depend on the microstructure, molecular weight, and isotopic labeling considered. The same applies for other parameters reported in the following, as, for example, the self-concentration or the characteristic time at the glass transition. Therefore, the values here obtained in these cases cannot be considered as characteristic for “generic” SBR and “generic” PS.

Blends of different compositions were prepared by solution casting using tetrahydrofuran (THF) as a solvent. The compositions of the mixtures of fully protonated SBR and deuterated PS were chosen such that the molar composition corresponded to mixtures of SBR and fully protonated PS with SBR weight fractions (*w*_SBR_) of 0.8, 0.6, 0.5, and 0.3. The obtained films were carefully dried under vacuum at 343 K for 24 h to remove the solvent completely. Reference samples of the neat polymers were prepared in a similar way.

### 3.2 Differential Scanning Calorimetry

DSC measurements were carried out on approximately 10 mg of samples using a Q2000 TA instruments. A liquid nitrogen cooling system (LNCS) was used with a 25 mL/min helium flow rate. Measurements were performed by placing the samples into aluminum pans. Data were acquired during cooling at 3 K/min from 353 K to 173 K. Temperature-modulated experiments (MDSC) were performed using a sinusoidal variation of 0.5 K amplitude and 60 s period.

### 3.3 Small-Angle Neutron Scattering

SANS experiments on the blends were performed on the instrument D22 at the Institute Laue-Lagevin (ILL) in Grenoble, France.^41^ Using an incident wavelength λ = 6 Å and sample–detector distances (SSDs) of 17, 5.6, and 1.5 m, a *Q*-range between 0.003 and 0.58 Å^–1^ was covered. Here, the modulus of the scattering vector *Q* is defined as *Q* = 4πλ^–1^ sin(θ/2), with θ as the scattering angle. The samples with a thickness of 1 mm were sandwiched between aluminum foils. Experiments were carried out in isothermal conditions at 265, 277, and 298 K. Equilibration times of about 45 min were employed at each temperature. The data were reduced using ILL in-house software, correcting measured intensities for the transmission, deadtime, sample background, and detector background (with B_4_C as a neutron absorber at the sample position).

### 3.4 Broad-Band Dielectric Spectroscopy

BDS experiments were conducted by using an Alpha dielectric analyzer (Novocontrol) to determine the complex dielectric permittivity (ε* = ε′ – *i*ε*″*) over the frequency range from 10^–2^ to 10^7^ Hz. Samples were placed between two flat gold-plated electrodes (30 and 20 mm diameter) forming a parallel plate capacitor with a 0.1 mm thick cross-shaped spacer of Teflon of negligible area between them. The temperature was controlled by a nitrogen jet-stream with a Novocontrol Quatro temperature controller. The measured temperature range was 130–360 K, and data were recorded every 5–10 K.

## 4 Results

### 4.1 Calorimetric Traces of the Glass Transition

**Figure 1:**
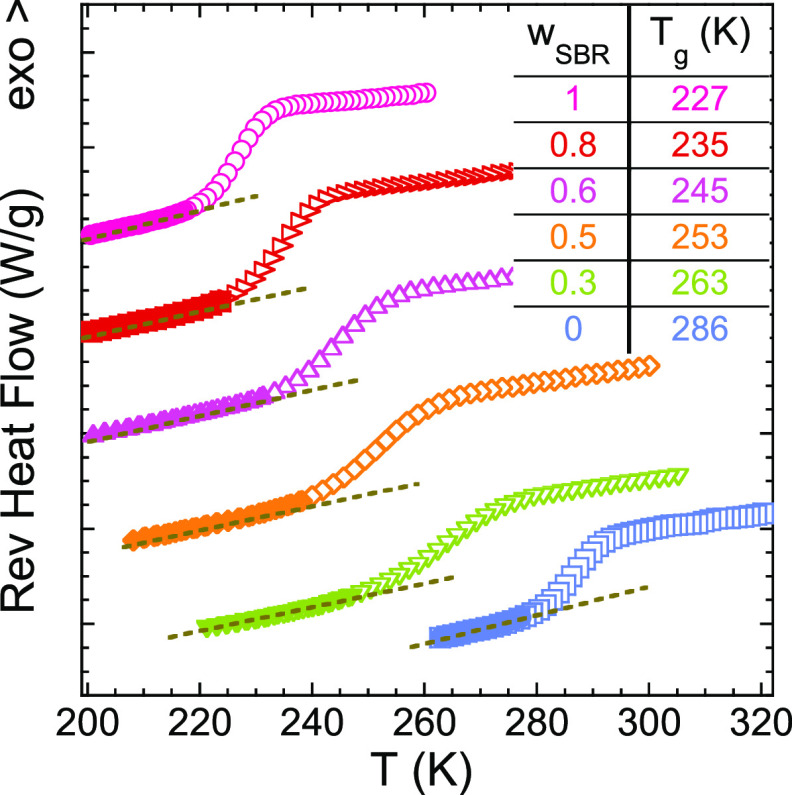
Reversible heat flow during cooling at 3 K/min for the pure components and SBR/PS blends. Data were vertically shifted for the sake of clarity. The composition and the glass transition temperatures are specified for each sample. The dashed lines correspond to the linear description of the glassy part.

The calorimetric *T*_g_ values of the samples were determined by picking up the inflection point of the reversible part of the heat flow during cooling at 3 K/min (see Figure 1). The difference between the *T*_g_s of the two neat systems, Δ*T*_g_s, is around 60 K, and their blends can be considered as dynamically asymmetric binary blends.^5^ In this system, PS (*T*_g_ = 286 K) is the high-*T*_g_ or slow component and SBR (*T*_g_ = 227 K) is the low-*T*_g_ or fast component. The glass-transition processes of the blends manifest broad features in the range between the *T*_g_s of the pure components; as we increase the content of PS, the heat flow jump range becomes broader.

**Figure 2:**
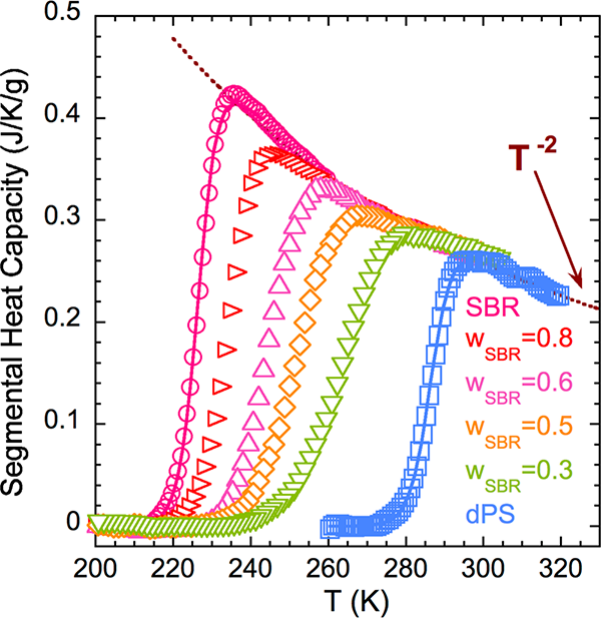
Calorimetric traces after the subtraction of the glassy part; same procedure has been applied on the neat components and the mixtures of SBR/PS. The solid lines fitting the neat polymers data were obtained by using eq 8 with parameters given in Table 2.

In order to analyze the contributions to the experimental DSC trace of the segmental dynamics responsible for the glass transition, first the glassy behavior has been accounted for with a linear function (for the sake of simplicity) and subtracted from the DSC cooling scan of the reversible heat flow (Figure 1). We have used this procedure for the homopolymers as well as for the blends. The resulting calorimetric traces that will be used for the following analysis are shown in Figure 2 and will be referred to as segmental heat capacity, s*-C*_*p*_. Interestingly enough, the behavior at temperatures well above *T*_g_ for all samples nearly superimposes and can be approximately described by a power law (*T*^*–n*^) with *n* = 2. It should be noted that the subtraction of a linear function does not alter the inflection point temperature determining *T*_g_.

It is a quite generally accepted that in bulk polymer systems the segmental dynamics fully controls the way thermal equilibrium is lost when decreasing temperature (the liquid to glass transition phenomenon). Despite the fact that several theoretical approaches exist,^42,44^ a fundamental quantitative link between segmental dynamics and the way thermodynamic equilibrium is lost has not been established by now. For instance, the Adam and Gibbs equation establishes a direct link between the characteristic times and the configurational entropy on the basis of the so-called cooperatively rearranging regions (CRRs).^42^ In this framework, as the temperature is reduced, the configurational entropy decreases and the time needed for maintaining thermodynamic equilibrium rapidly increases. In this way once the equilibration time exceeds typical laboratory values (ca. 1–1000 s, depending on the experimental conditions), equilibrium is lost and the supercooled liquid state transforms into a glassy state, the glass-transition phenomena. When comparing the relaxation times for the segmental dynamics and the calorimetric glass transition temperatures, semiquantitative connection can be established; namely, the relaxation time measured at the calorimetric *T*_g_ (taken as the inflection point) is on the order of 10 seconds.^45^

Taking this into consideration, the starting point of our approach is to first do a relatively simple full characterization of the homopolymers’ DSC behavior, which would encode the unsolved intricate connection between the segmental dynamics and the glass formation process. After that, we will use this simple picture to establish the connection between the segmental dynamics and the DSC data of the blends following a scheme mirroring that used before for the BDS data description.

The description of the DSC traces in the glass transition range for the neat polymers required quantifying the three main quantities for each component: a characteristic temperature, a measure of the width of the glass transition range, and the associated heat capacity jump. A simple but satisfactory way to describe the experimental segmental heat capacity of the neat polymers is by combining a sigmoidal function with a *T*^*–*2^ law as
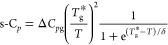
8where Δ*C*_*p*g_ is the heat capacity jump, δ measures the width of the glass transition range, and *T*_g_^*^ is a characteristic temperature defined as the inflection point of the sigmoidal function. As can be appreciated in Figure 2, the description of the experimental data for the neat components, both SBR and PS, is very good. The parameters determined by fitting the curves are given in Table 2. Note that minor differences exist between *T*_g_ defined as the inflection point of the full function and *T*_g_^*^ values. Nevertheless, the observed differences are close to the typical experimental uncertainties in determining *T*_g_ values.

**Table 2:** Parameters Obtained by Fitting Eq 8 to the Segmental Component of the Reversible Heat Flow of the Neat Components.

	δ/K	Δ*C*_*p*g_/J g^–1^ K^–1^	*T*_g_^*^/K
SBR	2.5	0.46	226.6
PS	2.6	0.28	286.5

The results presented in Figures 1 and 2 suggest a good miscibility (in the “traditional” meaning) of the SBR/PS blends in the full range of concentrations investigated, which will be confirmed in the following section from a thermodynamic point of view.

### 4.2 Analysis of the SANS Results

**Figure 3:**
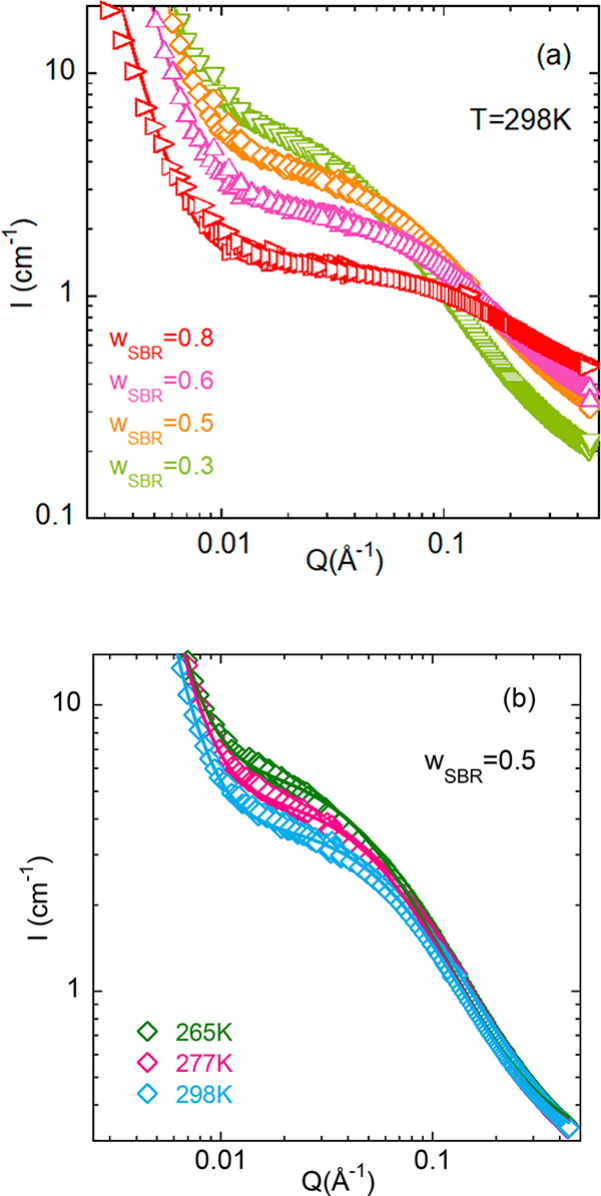
SANS results on the different SBR/PS blends at 298 K (a) and for *w*_SBR_ = 0.5 at the three temperatures investigated (b). Solid lines are fits using eq 10.

The DSC information—determining the supercooled liquid/glassy state boundaries—was combined with the SANS information on TCF, revealing the spinodal decomposition temperatures. Representative SANS results are shown in Figure 3a at 298 K for the different samples investigated and in Figure 3b for the sample with SBR content *w*_SBR_ = 0.5 as function of temperature. With decreasing *Q*, the data show a first clear increase of the scattered intensity followed by a plateau. This regime is dominated by TCFs in the mixture. The amplitude of this contribution strongly depends on composition. For a given sample, as shown in Figure 3b, the amplitude of TCFs increases with decreasing temperature. To characterize the TCFs the Ornstein–Zernike (OZ) expression is usually invoked:
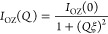
9where *I*_OZ_(0), the *Q* → 0 value of the function, is the amplitude and ξ is the correlation length for TCFs. The OZ function is in general a good approximation of the structure factor of polymer blends in the random phase approximation (RPA).^32−35^ Below *Q* ≈ 0.015 Å^–1^, an additional contribution to the scattered intensity is found, which varies as ∼*Q*^–*x*^ with *x* ≈ 4. The origin of this contribution to the scattering is controversial, and its interpretation is beyond the scope of our work. We just parametrize it with a Porod-like power law ∼*Q*^–4^ to properly obtain the information on the OZ contribution. In order to describe the SANS results, we also need to consider a background (BG), accounting for incoherent contributions. These are higher for samples richer in protonated component, i.e., with increasing SBR concentration. With all, the data were fitted by the following expression:
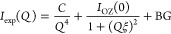
10Figure 3 shows that this kind of description works rather well.

**Figure 4:**
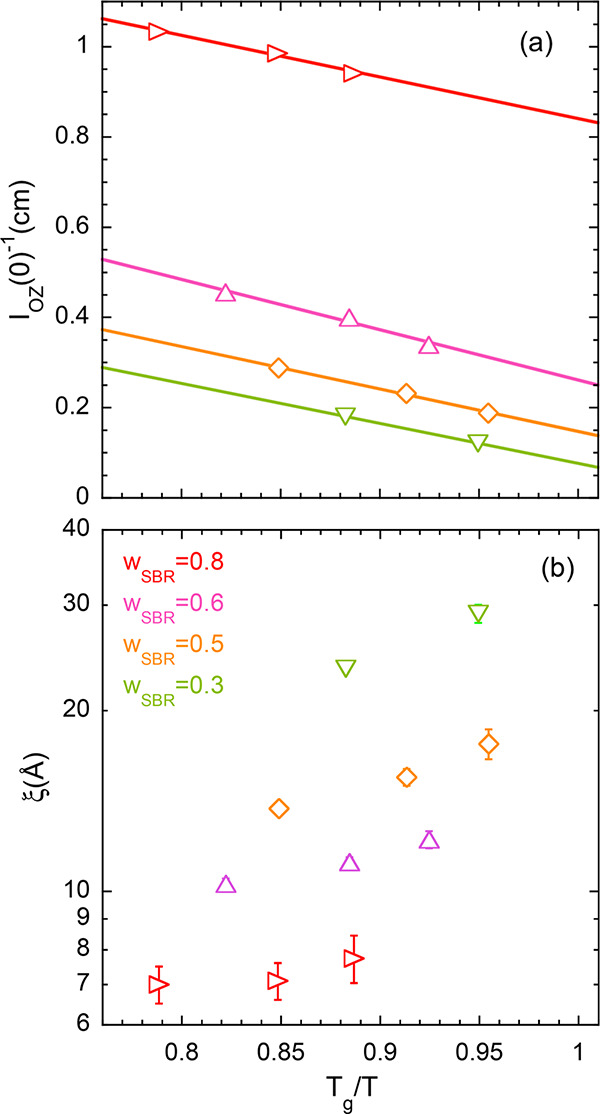
Inverse amplitudes (a) and correlation length (b) of the Ornstein–Zernike contribution to the SANS patterns as functions of *T*_g_/*T*, where *T*_g_ is the calorimetric average glass-transition temperature of the corresponding sample. Lines in (a) are linear fits. The code for the SBR weight fraction of the different blends is shown in (b).

**Figure 5:**
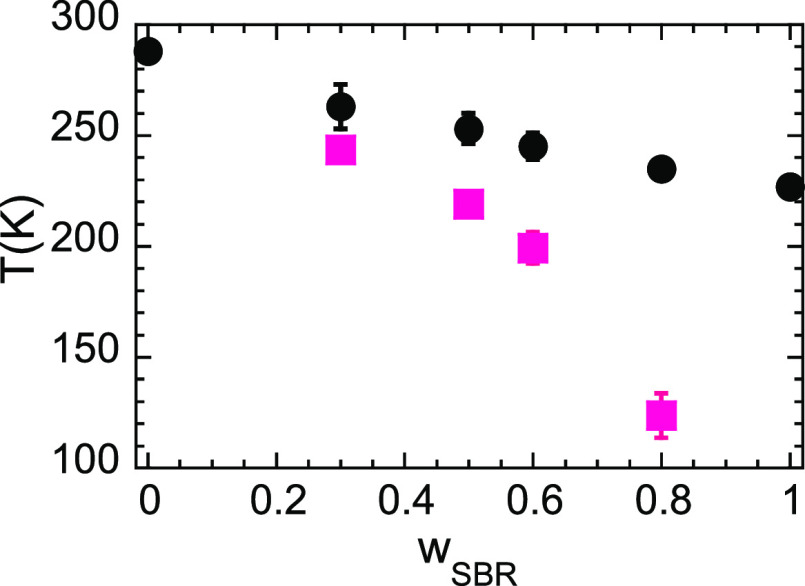
Average glass-transition temperature for the SBR/PS blends obtained from DSC (*T*_g_, circles) and spinodal decomposition temperature deduced from SANS (*T*_s_, squares). Bars on *T*_g_-values display the limits of the calorimetric glass transition range (initial and final temperatures of the heat flow step as seen in Figure 1), and bars on *T*_s_ data represent the estimated uncertainties in their determination.

To determine the spinodal decomposition temperature *T*_s_ (where the amplitude of TCF diverges) for the different compositions, we have represented in Figure 4a the inverse values of the OZ amplitudes *I*_OZ_(0) as function of the inverse temperature. Actually, we have plotted the results against the variable *T*_g_/*T*, where the *T*_g_ value has been previously determined as the inflection point of the DSC trace, to clearly discern whether the spinodal temperature is below or above the calorimetric average glass transition. Actually, we discarded the lowest temperature (265 K) results for the 30 wt % sample, because this temperature is very close to the average temperature of the blend and total equilibrium was not assured, even with the long equilibration time employed in the measurements. From Figure 4a we can see that the signatures of TCF are amplified with decreasing temperature. At the same time, the correlation length ξ increases (see Figure 4b). These observations point to phase separation of the mixtures at low temperatures (UCST-type phase behavior). The values of *T*_s_ were obtained as the intercept of the linear fit of the data in Figure 4a with the *x*-axis. These values are represented in Figure 5 together with the calorimetric results on the vitrification phenomenon. For all the compositions investigated, the value of *T*_g_ is always higher than *T*_s_: upon cooling, the sample becomes a glass before demixing. In other words, in the supercooled liquid regime the blends are stable mixtures from a thermodynamic point of view.

SANS results also allow determining the effective interaction parameter between components χ. The χ-values obtained (see Supporting Information, SI) are lower than those reported for blends of the same PS oligomers and SBR of different microstructure (lower styrene content, leading to a lower glass-transition temperature and thereby enhanced dynamic asymmetry).^31^ This result reflects an improved compatibility of the components when the copolymer has more chemical and dynamic similarity with the oligomer. On the other hand, we note that the analysis of the *Q*-dependence of our SANS results in terms of the habitual RPA^32,35^ is not trivial due to the oligomeric character of the PS component. This would require the description of the form factor for a finite number of monomers with some chain stiffness. This kind of analysis is beyond the scope of the present work.

### 4.3 Modeling the Dielectric Response with the Input of SANS

Once we confirmed that in the supercooled liquid regime the investigated mixtures were stable against phase separation, BDS experiments were performed and described using the simple model presented above; see the SI for details. The dielectric relaxation modeling was based on assuming that the relaxation shape and intensity of the neat components and those of these components inside each of the regions “*i*” forming the mixtures are the same. The dielectric α-relaxation of each neat component can be described by a Havriliak–Negami equation^46^ with temperature-independent shape parameters (see the SI). These characteristics are maintained for the contribution of the considered component to the relaxation of the blend, the relaxation time being the parameter assumed to be affected by blending.

**Figure 6:**
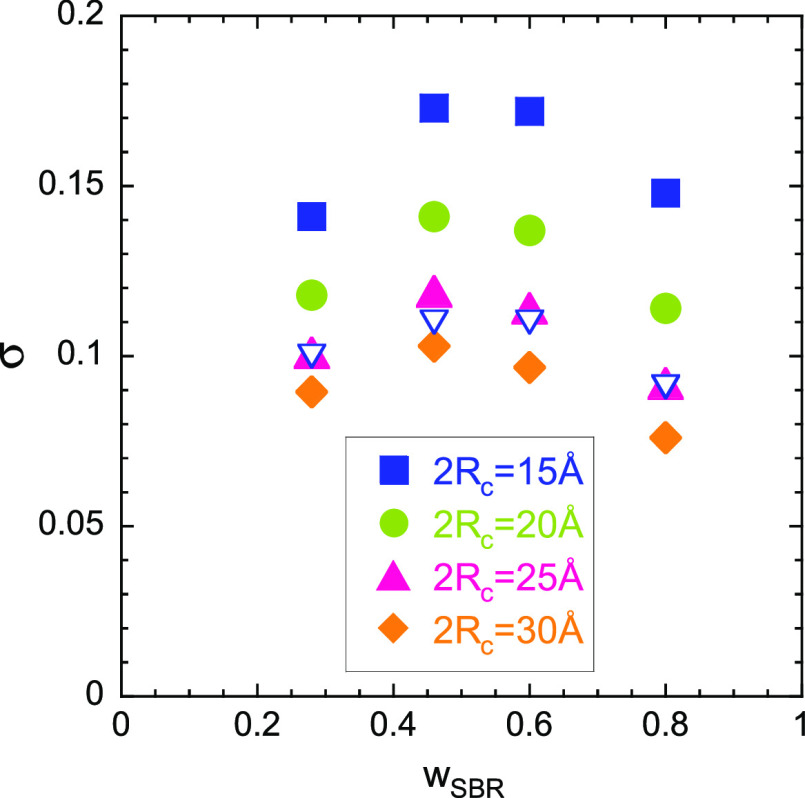
Concentration dependence of the width of the Gaussian distributions of concentration fluctuations deduced from the SANS results assuming different values for the relevant length scale 2*R*_*c*_ (filled symbols). Empty symbols correspond to values obtained by fitting BDS data (see Figure S4) without constraints, at temperatures where the dielectric loss peak is well centered in the experimental frequency window.

In this way, we have applied the modeling strategy to the BDS results in the blends (see the SI). The parameters involved are the self-concentrations of both components φ_self_^SBR^ and φ_self_^PS^ determining the local composition in each region and the widths σ of the distributions of concentration associated with the spontaneous fluctuations, described by means of Gaussian functions, *g*(*φ*_*i*_), eq 1. In previous works, the composition-dependent values of σ were obtained from fitting the BDS experimental results. However, with the information from SANS experiments that provide direct insight on the TCFs we can independently obtain information about σ. Based on previous works of Fischer et al.,^6,43^ Colby, Kumar, et al.^26^ proposed that in an incompressible binary blend the mean-squared concentration fluctuation σ^*2*^ is given by
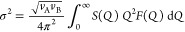
11where *v*_A_ and *v*_B_ are the monomeric volumes of the components A and B and *F*(*Q*) is the form factor of the considered volume. As discussed above, the segmental dynamics has a cooperative character and involves the correlated motion of many units. Thus, the relevant volume for calculating σ from SANS data would be directly connected to the region where the correlated motions occur. Nowadays there is increasing evidence that the correlated motions giving rise to the structural relaxation in the supercooled liquid regime occur in string-like entities.^47−49^ Moreover, it has been proposed very recently that the same entities could also be involved in other universal characteristics of the amorphous materials, particularly the so-called Boson peak.^49^ Despite these results, for the sake of maintaining the spirit of simplicity in our approach, and following previous works,^6,30,50^ the relevant volume for the segmental dynamics has been assumed to be a sphere of radius *R*_c_. Within this approach, if as in the present case an OZ function (see eq 9) is used to describe the structure factor *S*(*Q*), eq 11 can be expressed as
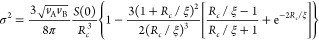
12Here *S*(0) = *I*_OZ_(0)/(Δρ)^2^, with Δρ being the difference in scattering length density of the two components. Introducing the values of *v*_A_, *v*_B_, and Δρ corresponding to our blend components (see the Experimental Section) and the values experimentally determined for *I*_OZ_(0) and ξ (Figure 4) we can calculate the concentration-dependent σ-values for a given value of the relevant length scale 2*R*_*c*_. Figure 6 shows the results obtained for some explored values of 2*R*_*c*_ using the SANS data at 298 K (the highest explored temperature). Despite the fact that *I*_OZ_(0) and ξ change very clearly with temperature in the temperature range investigated, for each value of 2*R*_*c*_, σ-values obtained at lower temperatures resulted to be very similar, within typical uncertainties (see Figure S8). Thus, in good approximation σ-values can be considered as temperature independent. Taking these results into account, we looked for the a priori unknown relevant length scale for the α-relaxation 2*R*_*c*_.

In a first step we analyzed representative BDS curves of the four mixtures allowing the three parameters φ_self_^SBR^, φ_self_^PS^, and σ to vary freely. In this way, we obtained composition-dependent σ-values that were compared with those deduced from SANS (see Figure 6). From this comparison it is clear that the obtained values from BDS are close to SANS values for 2*R*_*c*_ = 25 Å. Consequently, in a second step we fixed the σ-values to those obtained from SANS for 2*R*_*c*_ = 25 Å, namely (see Figure S9), σ_0.8_ = 0.09, σ_0.6_ = 0.11, σ_0.5_ = 0.115, and σ_0.3_ = 0.10 (where the subindex refers to the nominal SBR weight fraction). The new fitting of the BDS data was performed allowing φ_self_^SBR^ and φ_self_^PS^ to vary but imposing temperature- and composition-independent values as an additional condition. As can be seen in Figure S4 of the SI, φ_self_^SBR^ = 0.14 and φ_self_^PS^ = 0.19 allow an overall very satisfactory description of the BDS results. Therefore, this approach allows obtaining a good description of BDS data that is based on three temperature- and concentration-independent quantities, the two self-concentration values and the relevant length scale for the α-relaxation. Note that in the followed method the uncertainty in 2*R*_*c*_ is ±5 Å and the estimated uncertainties for SBR and PS self-concentration values were respectively ±0.04 and ±0.05.

When comparing the self-concentration values obtained by this analysis with literature results, we found that they are within the usual range.^16^ Nevertheless, the involved uncertainties prevent a detailed comparison. Moreover, it is noteworthy that the present approach involves many a priori assumptions, and the best values for the fitting parameters could be influenced by the limited validity of some of these assumptions. Concerning the relevant length scale for the α-relaxation, the value 2*R*_*c*_ = 25(±5) Å we have found is also in the nanometer range, which is, for instance, where evident confinement effects on the segmental dynamics have been reported.^51^ Concerning the possible increase of this length scale by reducing temperature,^52^ which is usually invoked for explaining the temperature dependence of the relaxation times, the uncertainties involved in our approach also prevent resolving it.

### 4.4 Connecting the Segmental Dynamics Modeling with the DSC Behavior of SBR/PS Blends

In an equivalent way to that followed for the BDS modeling, for the calorimetric description we will assume that the observed behavior is the result of the superposition of contributions to the segmental heat capacity from different regions and within each region the result of the individual contributions from the blend components. Thus, the whole calorimetric signal can be obtained by summing up the respective contributions of SBR and PS:
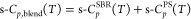
13

**Figure 7:**
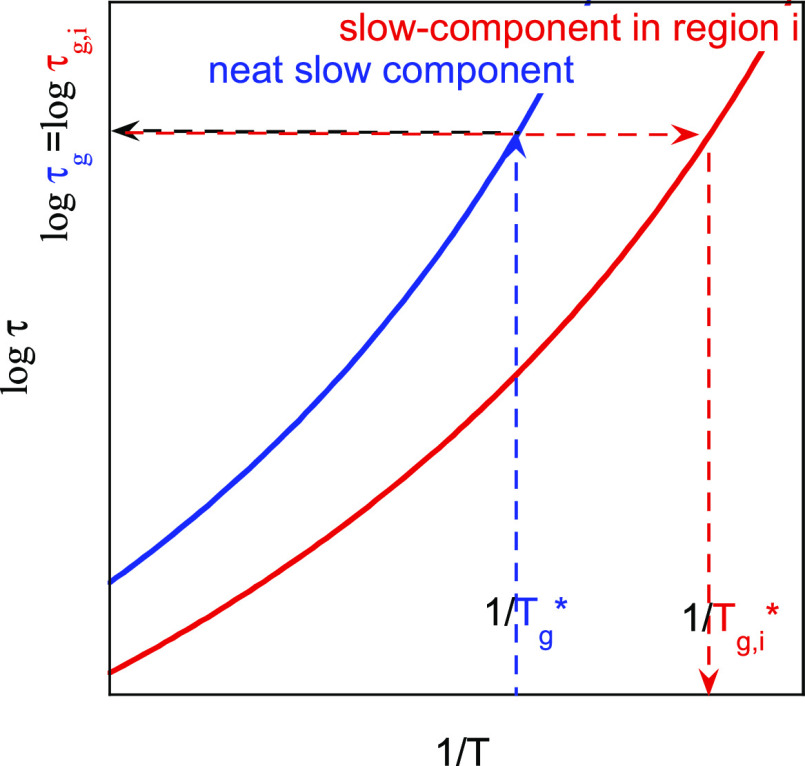
Schematics of the temperature dependence of the characteristic times of a neat component and the corresponding component in a given region of the blend. The lines with arrows show how the connection between BDS relaxation time data and the *T*_g_^*^-value is done.

Also, in parallel with the BDS modeling, the contribution of each component in a region *i* of the blend is taken having the shape and amplitude corresponding to the pure component and weighted by its concentration. Thus, the segmental heat capacity as a function of temperature for each component can be calculated as
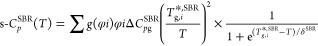
14a
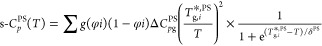
14bwhere we have assumed that in the description of the segmental heat capacity the only parameter affected by blending is *T*_g,i_^*^. This approach is in line with the BDS modeling where we considered that in each region in the blend only the relaxation time of the components is affected. The usual identification of the glass transition temperature with that where the characteristic time for polymer segmental motions takes a given value (commonly in the range 1–100 s) provides the way of connecting DSC and BDS modeling. Using the neat polymers DSC and BDS data allows us to connect the DSC *T*_g_^*^ value and the dielectric relaxation time evaluated at this temperature τ(*T*_g_^*^) for the two components. From the analysis of the data of the pure polymers SBR and PS (see the SI) we found, respectively, that the relationship between the dielectric α-relaxation time and the calorimetric *T*_g_^*^ is τ_g_^SBR^ ≡ τ^SBR^(*T*_g_^*^) = 1.68 s and τ_g_^PS^ ≡ τ^PS^(*T*_g_^*^) = 11.2 s. We will assume that these connections remain valid in each region of the blend, and in this way *T*_g,i_^*^ values appearing in eqs 14a and 14b can be calculated from the BDS modeling (Table 3). Figure 7 illustrates this assumption for the high-*T*_g_ component. From the calorimetric *T*_g_^*^ of the pure polymer, the dielectric relaxation time *τ*_g_≡ τ(*T*_g_^*^) is first evaluated as above-mentioned (blue arrows). Afterward, the temperature *T*_g,i_^*^ where the same component located in a given region *i* of the blend contributes to the heat capacity jump is calculated from the corresponding relaxation time curve as obtained by the dielectric modeling (red arrows). This means that, in the present approach, for each component there is a one-to-one correspondence between the local dielectric relaxation time characterizing the segmental dynamics and the glass transition temperature determining the temperature range where the thermodynamic local equilibrium is lost.

**Table 3:** Parameters Involved in the Description of the Dielectric α-Relaxation of the Blends That Are Also Relevant for the Corresponding Description of the Calorimetric Data

sample	*D*	*T*_0_/K	τ_max_(*T*_g_^*^)/s	φ_self_
SBR	8.6	176.7	1.68	0.14
PS	6.3	239.8	11.2	0.19

**Figure 8:**
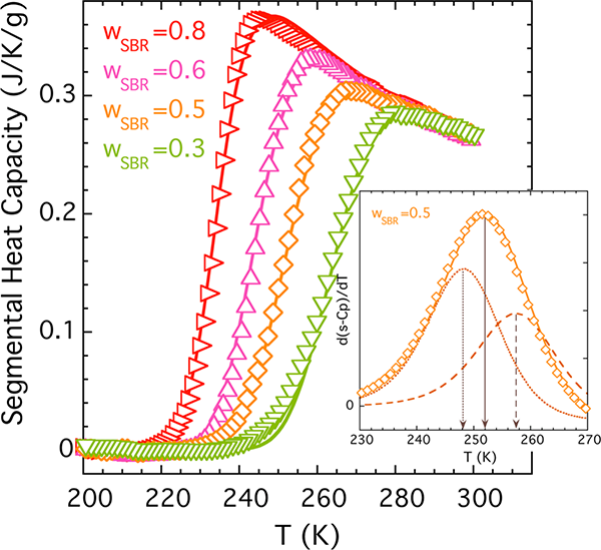
Segmental heat capacity for SBR/PS blends with the indicated compositions. Solid lines stand for the output of the model described in the text. Inset: temperature derivative of the segmental heat capacity and corresponding output model (solid line) for the *w*_SBR_ = 0.5 blend; dotted and dashed lines show respectively the model contributions of SBR and PS components. Vertical lines illustrate how the corresponding values of *T*_g_ are evaluated.

After establishing this connection between the local segmental dynamics of each component and the glass transition temperature, the DSC curves were evaluated using the model describing the BDS results (see the SI). For the DSC calculations the local composition was described in terms of the same values of the self-concentrations φ_self_^SBR^ and φ_self_^PS^ (Table 3) and the distributions of concentration by means of same Gaussian functions *g*(φ_*i*_) with the σ-values obtained from the SANS results, i.e., with 2*R*_*c*_ ≡ 25Å: σ_0.8_ = 0.09, σ_0.6_ = 0.11, σ_0.5_ = 0.115, and σ_0.3_ = 0.10. Thus, the evaluation of the DSC curves for the blends can be made with no additional free parameters. The resulting curves are shown in Figure 8 in comparison with the experimental data for the different blends investigated, where an overall excellent agreement between the two sets of data can be observed. In the particular case of the blend with *w*_SBR_ = 0.5 the individual contributions from SBR and PS components (see eq 13) have also been presented in the inset as dotted and dashed lines, respectively.

## 5 Discussion

In the previous section we have shown how the simple model previously used to describe simultaneously the dielectric and mechanical relaxation of industrial simplified mixtures of SBR with a PS oligomer (up to *w*_SBR_ = 0.5)^30^ can also be extended to the full composition range. Furthermore, based on the same scheme we develop a way of connecting the segmental dynamics of the mixtures with the DSC experiments. The calculated behavior in this way provides a very good description of the experimental DSC curves of this kind of systems. Noteworthy, this approach is based on only three temperature- and composition-independent parameters, in addition to those required for the description of the neat components. The microscopic insight provided by SANS has made it possible to eliminate the freedom on the concentration dependence of the width of the distribution of TCFs used in previous works.^29,30^ In the way we have approached now the modeling of the α-relaxation, the only unknown parameter involved in the characterization of the TCFs is the relevant length scale of segmental relaxation. Once this is fixed, from SANS we can independently deduce the values of the widths of the distributions of TCFs and impose them in the description of the BDS results. The value obtained for the relevant length scale, ∼25 Å, is close to that deduced by us in a recent work on blends of the same PS-oligomers and a lower molecular weight SBR of different microstructure, invoking the same framework.^31^ Thus, as the respective self-concentration parameters of the components were taken as concentration independent, the number of free parameters accounting for blending effects involved in the BDS description is, effectively, only three: φ_self_^SBR^, φ_self_^PS^, and 2*R*_*c*_. Despite the various assumptions and simplifications involved in our approach, the parameter values we obtained are in the range one could expect on the basis of the fundamental understanding of the polymer segmental dynamics.^5^

The presented modeling not only provides a good description of the BDS data characterizing the segmental dynamics of the blends at equilibrium but also, without extra variables, allows obtaining the DSC behavior reflecting how thermodynamic equilibrium is lost when cooling the mixtures below the glass-transition range.

**Figure 9:**
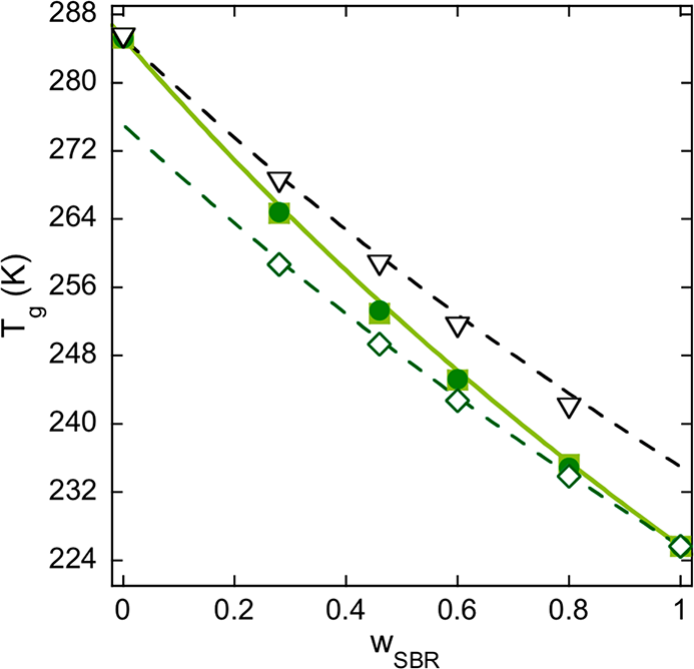
Comparison of the concentration dependence of the glass transition temperature as determined from the experimental curves (filled squares), the whole model curve (filled circles), and the effective values from the model curves of the components (inverted empty triangles for PS and empty diamonds for SBR). The lines are the prediction of the Fox equation (eq 15) for the blends (solid line) and for the components using the model φ_self_ values (dashed lines) and calculated with eqs 16.

As a further test of the ability of the model in accounting for the calorimetric behavior, Figure 9 shows the direct comparison between experimental and calculated values of *T*_g_ as a function of blend composition, both series calculated from the inflection point of the segmental heat capacity s*-C*_*p*_(*T*) curves (peak temperatures in Figure S5). A very good agreement is obtained in this comparison. Interestingly, even if the SANS results showed that this blend system is not athermal, the whole set of data is very well described by the Fox equation:^21^
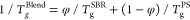
15

As shown before, the modeling provides not only the overall DSC curves but also the individual contributions from SBR and PS components (see, for example, dotted and dashed lines in the inset of Figure 8). From the inflection point of the thus calculated s*-C*_*p*_(*T*) curves for the components, the so-called effective glass-transition temperature^16^ can be defined for each component of the blend. These effective *T*_g,eff_ values have been included in Figure 9. Here we can see that they can also be very well described with the Fox-like equation (see dashed lines in Figure 9) by using the effective concentration as calculated with the same self-concentration values deduced from the modeling of the BDS results, i.e.,
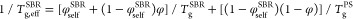
16a
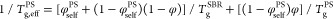
16b

Note that these two equations are formally similar to eqs 6a and 6b used in the model when evaluating the values of *T*_0,*i*_ as a function of the local concentration, but here we evaluate the overall *T*_g,eff_ values of each component.

The presented results suggest that the loss of equilibrium in a polymer blend as detected by the DSC glass-transition phenomenon can be accounted for by the simple superposition of contributions, each following a neat-polymer-like behavior but occurring at different temperatures depending on the local concentration around the considered component. Thus, in every region of the blend the equilibrium is lost in a way that can be considered as independent of the neighboring regions. In addition, the glassy state would be reached in two steps within each region, each associated with one of the components.

The very satisfactory description of the evolution of the *T*_*g*_ values (global and effective) with composition evidences that, despite the simplicity of the present approach, it correctly captures the connection of the component segmental dynamics in the blend above *T*_g_ (at equilibrium) and the way the thermodynamic equilibrium is lost when crossing over to the glassy state. This is to some extent surprising, since the model of the dielectric curves was built to describe the equilibrium dynamics, and the connection between the equilibrium dynamics and how the equilibrium is lost below *T*_g_ is made in a very phenomenological way, using only the individual polymer component for parametrization. The results suggest that the τ_g_ = τ(*T*_g_^*^) is an intrinsic magnitude of a given material, which is not affected by blending.

The comparison of SANS and BDS results has allowed establishing the relevant length scale for the segmental relaxation in these blends, as monitored by dielectric spectroscopy. The excellent agreement also with the DSC traces shows that this would also be the relevant length scale for the glass-formation process. The value found—2*R*_*c*_ ≈ 25Å—is in the range usually assumed for the α-relaxation in many glass-forming systems.^37^ Thus, with this work we provide additional experimental support to the relevance of nanometric length scales in the vitrification phenomenon. We note that 2*R*_*c*_ and ξ are completely independent magnitudes, the former related to the dynamics of the α-relaxation associated with the glass-forming character of the material, and the latter arising exclusively in mixtures.

Taking into account the good quality of the description of the DSC experiments using this simplified approach, it would be eventually possible to extract the model parameters just by using the DSC data of the blends. In such case, the dynamical properties of the mixture could be anticipated from those of the pure components based on rather routine experiments on the blends. Exploring this possibility will be the subject of our future work in this context.

## 6 Conclusions

We have investigated the thermodynamic, dynamic, and structural properties of a simplified system of industrial interest consisting of blends of SBR and PS-oligomers. To this end, three techniques, DSC, BDS, and SANS, have been applied. A model based on the combination of two main ingredients, namely, the self-concentration^16^ and the thermally driven concentration fluctuations,^5,6,8,11,19,20^ has been invoked to account for the effects of blending. This model had been applied until now to describe the equilibrium dynamics as monitored by BDS and mechanical spectroscopies.^29,30^ The main contributions of this work are (i) to use the microscopic SANS insight into TCF to fully characterize their impact on the broadening of the segmental dynamics observed by BDS and (ii) to connect the modeling of the segmental dynamics of the blends with the individual contributions of the blend components to the DSC behavior. Thanks to this input, we have been able to successfully describe blending effects not only on the equilibrium dynamics of the α-relaxation in the miscible state as monitored by BDS but also on the DSC manifestation of the glass-transition phenomenon reflecting how thermodynamic equilibrium is lost, by using only three free parameters: the self-concentration of the components and the relevant length scale of segmental relaxation. This approach reproduces very well the experimental results in a wide range of temperatures and compositions, supporting the validity of the rather rough assumptions involved. The characteristic time of each component evaluated at the glass-transition temperature is apparently not affected by blending. In addition, the present approach allows decomposing the DSC result into the component contributions, providing the composition-dependent values of the effective glass transition temperatures. They can be consistently described by the standard Fox equation using the self-concentration values, giving additional support to our framework. Further support is provided by the excellent agreement obtained invoking the same effect of TCF on both BDS and DSC, pointing to the same relevant length scale for the dynamics of the α-relaxation and the loss of equilibrium at the glass transition. This length scale is about 2.5 nm, in accordance with previous works on glass-forming systems.^37^ Moreover, the values of self-concentration found are in the range one could expect for this type of polymers for such length scale, which gives basic support for the various approximations involved in the modeling.

## Supporting Information Available

(i) Dielectric relaxation of neat components; (ii) dielectric α-relaxation of SBR/PS blends; (iii) derivative of the calorimetric traces of SBR/PS blends; (iv) determination of the effective interaction parameter from SANS; (v) temperature dependence of the width of concentration fluctuations (PDF)

## References

[ref1] Utracki L.; Wilkie C., Eds.; Polymer Blends Handbook; Springer Netherlands, 2014.

[ref2] Isayev A. I., Ed. Encyclopedia of Polymer Blends; Wiley, 2010; Vol. 1.

[ref3] Silva G. G.; Machado J. C.; Song M.; Hourston D. J. Nanoheterogeneities in PEO/PMMA Blends: A Modulated Differential Scanning Calorimetry Approach. J. Appl. Polym. Sci. 2000, 77, 2034–2043. 10.1002/1097-4628(20000829)77:9<2034::AID-APP20>3.0.CO;2-Q.

[ref4] Lodge T. P.; Wood E. R.; Haley J. C. Two Calorimetric Glass Transitions Do Not Necessarily Indicate Immiscibility: The Case Of PEO/PMMA. Journal of Polymer Science Part B: Polymer Physics 2006, 44, 756–763. 10.1002/polb.20735.

[ref5] Colmenero J.; Arbe A. Segmental Dynamics in Miscible Polymer Blends: Recent Results and Open Questions. Soft Matter 2007, 3, 1474–1485. 10.1039/b710141d. 32900101

[ref6] Zetsche A.; Fischer E. W. Dielectric Studies of the α-Relaxation in Miscible Polymer Blends and its Relation to Concentration Fluctuations. Acta Polym. 1994, 45, 168–175. 10.1002/actp.1994.010450306.

[ref7] Cendoya I.; Alegría A.; Alberdi J. M.; Colmenero J.; Grimm H.; Richter D.; Frick B. Effect of Blending on the PVME Dynamics. A Dielectric, NMR, and QENS Investigation. Macromolecules 1999, 32, 4065–4078. 10.1021/ma9819539.

[ref8] Lutz T. R.; He Y.; Ediger M. D.; Cao H.; Lin G.; Jones A. A. Rapid Poly(ethylene oxide) Segmental Dynamics in Blends with Poly(methyl methacrylate). Macromolecules 2003, 36, 1724–1730. 10.1021/ma021634o.

[ref9] Leroy E.; Alegría A.; Colmenero J. Quantitative Study of Chain Connectivity Inducing Effective Glass Transition Temperatures in Miscible Polymer Blends. Macromolecules 2002, 17, 5587–5590. 10.1021/ma025508w.

[ref10] Alegría A.; Colmenero J.; Ngai K. L.; Roland C. M. Observation of the Component Dynamics in a Miscible Polymer Blend by Dielectric and Mechanical Spectroscopies. Macromolecules 1994, 27, 4486–4492. 10.1021/ma00094a009.

[ref11] Chung G. C.; Kornfield J. A.; Smith S. D. Component Dynamics Miscible Polymer Blends: A Two-Dimensional Deuteron NMR Investigation. Macromolecules 1994, 27, 964–973. 10.1021/ma00082a013.

[ref12] Genix A.-C.; Arbe A.; Arrese-Igor S.; Colmenero J.; Richter D.; Frick B.; Deen P. P. Neutron Scattering Investigation of a Diluted Blend of Poly(Ethylene Oxide) in Polyethersulfone. The Journal of Chemical Physics 2008, 128, 184901 10.1063/1.2918497. 18532842

[ref13] Arbe A.; Alegría A.; Colmenero J.; Hoffmann S.; Willner L.; Richter D. Segmental Dynamics in Poly(vinylethylene)/Polyisoprene Miscible Blends Revisited. A Neutron Scattering and Broad-Band Dielectric Spectroscopy Investigation. Macromolecules 1999, 32, 7572–7581. 10.1021/ma990402v.

[ref14] Schwahn D.; Pipich V.; Richter D. Composition and Long-Range Density Fluctuations in PEO/PMMA Polymer Blends: A Result of Asymmetric Component Mobility. Macromolecules 2012, 45, 2035–2049. 10.1021/ma2019123.

[ref15] Saiter J. M.; Grenet J.; Dargent E.; Saiter A.; Delbreilh L. Glass Transition Temperature and Value of the Relaxation Time at T_g_ in Vitreous Polymers. Macromol. Symp. 2007, 258, 152–161. 10.1002/masy.200751217.

[ref16] Lodge T. P.; McLeish T. C. B. Self-Concentrations and Effective Glass Transition Temperatures in Polymer Blends. Macromolecules 2000, 33, 5278–5284. 10.1021/ma9921706.

[ref17] Cangialosi D.; Alegría A.; Colmenero J. “Self-concentration” Effects on the Dynamics of a Polychlorinated Biphenyl Diluted in 1,4-Polybutadiene. The Journal of Chemical Physics 2007, 126, 204904 10.1063/1.2740632. 17552797

[ref18] Painter P.; Coleman M. Self-Contacts, Self-Concentration, and the Composition Dependence of the Glass Transition Temperature in Polymer Mixtures. Macromolecules 2009, 42, 820–829. 10.1021/ma802003p.

[ref19] Kumar S. K.; Colby R. H.; Anastasiadis S. H.; Fytas G. Concentration Fluctuation Induced Dynamic Heterogeneities in Polymer Blends. The Journal of Chemical Physics 1996, 105, 3777–3788. 10.1063/1.472198.

[ref20] Leroy E.; Alegría A.; Colmenero J. Segmental Dynamics in Miscible Polymer Blends: Modeling the Combined Effects of Chain Connectivity and Concentration Fluctuations. Macromolecules 2003, 19, 7280–7288. 10.1021/ma034144k.

[ref21] Fox T. G. Influence of Diluent and of Copolymer Composition on the Glass Temperature of a Polymer System. Bull. Am. Phys. Soc. 1956, 1, 123.

[ref22] Di Marzio E. A. The Glass Temperature of Polymer Blends. Polymer 1990, 31, 2294–2298. 10.1016/0032-3861(90)90315-P.

[ref23] Kwei T. K. The Effect of Hydrogen Bonding on The Glass Transition Temperatures of Polymer Mixtures. J.Polym. Sci.: Polym. Lett. Ed. 1984, 22, 307–313.

[ref24] Lin A. A.; Kwei T. K.; Reiser A. On The Physical Meaning of The Kwei Equation for the Glass Transition Temperature of Polymer Blends. Macromolecules 1989, 22, 4112–4119. 10.1021/ma00200a052.

[ref25] Lipson J. E. G. Global and Local Views of the Glass Transition in Mixtures. Macromolecules 2020, 53, 7219–7223. 10.1021/acs.macromol.0c01455.

[ref26] Shenogin S.; Kant R.; Colby R. H.; Kumar S. K. Dynamics of Miscible Polymer Blends: Predicting the Dielectric Response. Macromolecules 2007, 40, 5767–5775. 10.1021/ma070503q.

[ref27] Gaikwad A. N.; Wood E. R.; Ngai T.; Lodge T. P. Two Calorimetric Glass Transitions in Miscible Blends Containing Poly(ethylene oxide). Macromolecules 2008, 41, 2502–2508. 10.1021/ma702429r.

[ref28] Shi P.; Schach R.; Munch E.; Montes H.; Lequeux F. Glass Transition Distribution in Miscible Polymer Blends: From Calorimetry to Rheology. Macromolecules 2013, 46, 3611–3620. 10.1021/ma400417f.

[ref29] Gambino T.; Alegría A.; Arbe A.; Colmenero J.; Malicki N.; Dronet S.; Schnell B.; Lohstroh W.; Nemkovski K. Applying Polymer Blend Dynamics Concepts to a Simplified Industrial System. A Combined Effort by Dielectric Spectroscopy and Neutron Scattering. Macromolecules 2018, 51, 6692–6706. 10.1021/acs.macromol.8b00881.

[ref30] Gambino T.; Alegría A.; Arbe A.; Colmenero J.; Malicki N.; Dronet S. Modeling the High Frequency Mechanical Relaxation of Simplified Industrial Polymer Mixtures Using Dielectric Relaxation Results. Polymer 2020, 187, 122051 10.1016/j.polymer.2019.122051.

[ref31] Gambino T.; Shafqat N.; Alegria A.; Malicki N.; Dronet S.; Radulescu A.; Nemkovski K.; Arbe A.; Colmenero J. Concentration Fluctuations and Nanosegregation in a Simplified Industrial Blend with Large Dynamic Asymmetry. Macromolecules 2020, 53, 7150–7160. 10.1021/acs.macromol.0c01376.

[ref32] Higgins J. S.; Benoit H. C. Polymers and Neutron Scattering; Oxford University Press: Oxford, 1994.

[ref33] Rubinstein M.; Colby R. H. Polymer Physics; Oxford University Press: Oxford, U.K., 2003; Vol. 23.

[ref34] Wignall G. D.; Melnichenko Y. B. Recent Applications of Small-Angle Neutron Scattering in Strongly Interacting Soft Condensed Matter. Rep. Prog. Phys. 2005, 68, 1761–1810. 10.1088/0034-4885/68/8/R02.

[ref35] Mortensen K. Characterization of Polymer Blends; Wiley-VCH Verlag GmbH & Co. KGaA, 2014; pp 237–268.

[ref36] Rijal B.; Delbreilh L.; Saiter A. Dynamic Heterogeneity and Cooperative Length Scale at Dynamic Glass Transition in Glass Forming Liquids. Macromolecules 2015, 48, 8219–8231. 10.1021/acs.macromol.5b01152.

[ref37] Berthier L.; Biroli G.; Bouchaud J.-P.; Cipelletti L.; Masri D. E.; L’Hôte D.; Ladieu F.; Pierno M. Direct Experimental Evidence of a Growing Length Scale Accompanying the Glass Transition. Science 2005, 310, 1797–1800. 10.1126/science.1120714. 16357256

[ref38] Vogel H. The Law of the Relation between the Viscosity of Liquids and the Temperature. Phys. Z. 1921, 22, 645.

[ref39] Fulcher G. S. Analysis of Recent Measurements of the Viscosity of Glasses. J. Am. Ceram. Soc. 1925, 8, 339–355. 10.1111/j.1151-2916.1925.tb16731.x.

[ref40] Tammann G.; Hesse W. Die Abhängigkeit der Viscosität von der Temperatur bei unterkühlten Flüssigkeiten. Zeitschrift für anorganische und allgemeine Chemie 1926, 156, 245–257. 10.1002/zaac.19261560121.

[ref41] Moreno A.; Arbe A.; Colmenero J.; Formanek M.; Malo de Molina P.; Paciola M.; Pomposo J. A.; Porcar L.; Shafqat N. Conformation of single-chain nano-particles surrounded by linear chains: reversible vs irreversible bonds; Institut Laue-Langevin (ILL), 2020, 10.5291/ILL-DATA.9-11-1938.

[ref42] Adam G.; Gibbs J. H. On the temperature dependence of cooperative relaxation properties in glass-forming liquids. J. Chem. Phys. 1965, 43, 139–146. 10.1063/1.1696442.

[ref43] Lubchenko V.; Wolynes P. G. Theory of structural glasses and supercooled liquids. Annu. Rev. Phys. Chem. 2007, 58, 235–266. 10.1146/annurev.physchem.58.032806.104653. 17067282

[ref44] Dudowicz J.; Freed K. F.; Douglas J. F. Generalized entropy theory of polymer glass formation. Adv. Chem. Phys. 2007, 137, 125–222.

[ref45] Saiter J. M.; Grenet J.; Dargent E.; Saiter A; Delbreilh L. Glass Transition Temperature and Value of the Relaxation Time at Tg in Vitreous Polymers. Macromol. Symp. 2007, 258, 152–161. 10.1002/masy.200751217.

[ref46] Alvarez F.; Alegría A.; Colmenero J. Interconnection between Frequency-Domain Havriliak-Negami and Time-Domain Kohlrausch-Williams-Watts Relaxation Functions. Physical Review B 1993, 47, 125–130. 10.1103/PhysRevB.47.125. 10004424

[ref47] Starr F. W.; Douglas J. F.; Sastry S. The relationship of dynamical heterogeneity to the Adam-Gibbs and random first-order transition theories of glass formation. J. Chem. Phys. 2013, 138, 12A541 10.1063/1.4790138. PMC3598772 23556792

[ref48] Hung J-H.; Simmons Do String-like Cooperative Motions Predict Relaxation Times in Glass-Forming Liquids?. J. Phys. Chem. B 2020, 124, 266–276. 10.1021/acs.jpcb.9b09468. 31886663

[ref49] Hu Y. C.; Tanaka H. Origin of the boson peak in amorphous solids. Nature Physics 2022, 18, 669–677. 10.1038/s41567-022-01628-6.

[ref50] Katana G.; Fischer E. W.; Hack T.; Abetz V.; Kremer F. Influence of Concentration Fluctuations on the Dielectric α-Relaxation in Homogeneous Polymer Mixtures. Macromolecules 1995, 28, 2714–2722. 10.1021/ma00112a017.

[ref51] Alegria A.; Colmenero J. Dielectric relaxation of polymers: segmental dynamics under structural constrains. Soft Matter 2016, 12, 7709–7725. 10.1039/C6SM01298A. 27560167

[ref52] Donth E. The size of cooperatively rearranging regions at the glass transition. J. Non-Cryst. Solids 1982, 53, 325–330. 10.1016/0022-3093(82)90089-8.

