# Supplementary material for: Disentangling the Calorimetric Glass-Transition Trace in Polymer/Oligomer Mixtures from the Modeling of Dielectric Relaxation and the Input of Small-Angle Neutron Scattering
Source: Macromolecules. 2022 Aug 22;55(17):7614–25. doi: 10.1021/acs.macromol.2c00609 (PMC9477097; doi:10.1021/acs.macromol.2c00609)
Supplement: Supplementary file 1 — ma2c00609_si_001.pdf [file ma2c00609_si_001.pdf]

Supporting Information:

Disentangling the Calorimetric Glass Transition Trace in Polymer/Oligomer Mixtures from the Modeling of Dielectric Relaxation and the Input of Small Angle Neutron Scattering

Numera Shafqat[1,2], *Angel Alegría*[1,3,*], *Arantxa Arbe*[1], *Nicolas Malicki*[2], *Séverin Dronet*[2], *Lionel Porcar*[4], *and Juan Colmenero*[1,3,5]

[1]Materials Physics Center (MPC), Centro de Física de Materiales (CSIC, UPV/EHU), Paseo Manuel de Lardizabal 5, 20018 San Sebastián, Spain

[2]Manufacture Française des Pneumatiques MICHELIN, Site de Ladoux, 23 place des Carmes Déchaux, Cedex 63040 Clermont-Ferrand, France

[3]Departamento de Polímeros y Materiales Avanzados: Física, Química y Tecnología (UPV/EHU), Facultad de Química, 20018 San Sebastián, Spain

[4]Institut Laue-Langevin, 71 avenue des Martyrs, Grenoble Cedex 9, 38042, France

[5]Donostia International Physics Center, Paseo Manuel de Lardizabal 4, 20018 San Sebastián, Spain

*E-mail: angel.alegria@ehu.eus

The approach proposed here to calculate the DSC behavior in the SBR/PS blends is based on a direct connection between DSC and BDS experiments. Therefore, a previous simple modeling of the BDS experiments on the very same blends following a previously developed approach is required. Furthermore, this modeling is first based on the simple description of the dielectric relaxation of the neat components. Ergo in this section we first analyze the dielectric relaxation of the neat components and afterwards we use the already developed approach for modeling the dielectric relaxation data of the blends. In addition, we include some information from the SANS investigation.

(i) Dielectric relaxation of neat components

Figure S1 shows an example of the dielectric loss as a function of the frequency for the two pure polymers, SBR and PS, at temperatures where the main peak is well-centered in the experimental frequency window. In this representation we have used $Tan\ \delta \equiv \frac{\varepsilon''}{\varepsilon'}$ to minimize the impact of sample geometry changes that could occur for measurements over a large temperature interval. The main loss peak is due to the segmental dynamics or α-relaxations. SBR has a stronger dielectric relaxation than PS, it shows a larger loss peak area but both contributions will be relevant for the dielectric relaxation of the mixtures.

A full but simple characterization of the dielectric response of the pure polymers has been done first. The dielectric segmental α-relaxations can be described by means of the Havriliak-Negami (HN) equation: [1]

$$\varepsilon_{HN}^{*} = \varepsilon_{\infty} + \frac{\Delta\varepsilon}{(1+(i\omega\tau_{HN})^{\alpha})^{\gamma}} \qquad \text{(S1)}$$

where $\varepsilon_{\square}$ corresponds to the high-frequency limiting value of the permittivity, Δε is the dielectric relaxation strength, τ_{HN} is the characteristic relaxation time, α and γ are the fractional shape parameters describing the symmetric and asymmetric broadening of the complex dielectric function, maintaining the condition $0 < \gamma$ and $\alpha \cdot \gamma \leq 1$.

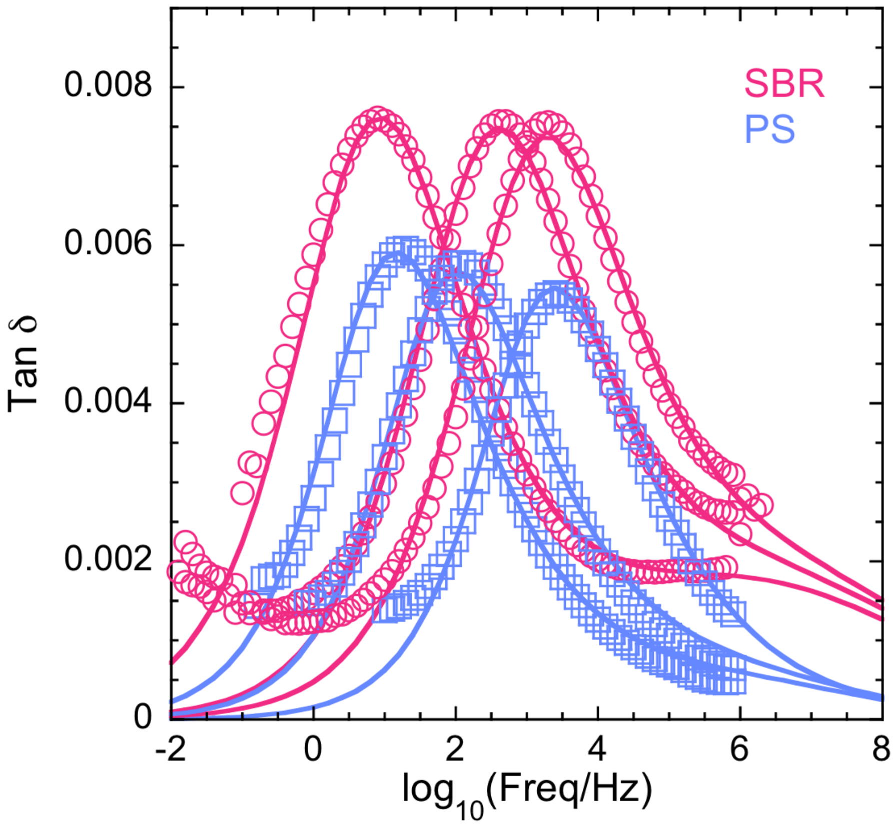

Figure S1. Frequency dependence of the dielectric Tan δ at 235K, 245K, and 250K for SBR (empty circles) and at 300K, 305K, and 315K for PS (empty squares). The solid lines represent the fits by means of the Havriliak-Negami equation for the α-relaxation and the addition of the β-relaxation calculated by extrapolating the lower temperature description.

In order to improve the description of the experimental data of the neat components, for both SBR and PS the contribution of the β-relaxation has been taken in account, which occurs at low-temperatures/high-frequencies. The dielectric losses corresponding to the β-relaxation can be fitted to a good approximation with a Gaussian function:

$$\varepsilon_{\beta}^{''} = A_{\beta} exp\left[-\frac{1}{2}\left(\frac{log_{10}(\omega\tau_{\beta})}{\sigma_{\beta}}\right)^{2}\right] \tag{S2}$$

Where A_{β} is the amplitude, τ_{β} is the relaxation time, and σ_{β} is a parameter accounting for the broadness of the peak. They are temperature dependent parameters, which were determined by fitting experimental data below T_g. From the analysis of the temperature dependent parameter for SBR we obtained:

$$\tau_{\beta}(T) = 5.41 \cdot 10^{-15}\, exp\left[\frac{3898\,K}{T}\right] \tag{S3a}$$

$$\sigma_{\beta}(T) = 1.5984 + \left(\frac{174\,K}{T}\right) \tag{S3b}$$

$$A_{\beta}(T) = 0.0024533 - \left(\frac{0.27784\,K}{T}\right) \tag{S3c}$$

While for PS we obtained:

$$\tau_{\beta}(T) = 1.50 \cdot 10^{-9}\, exp\left[\frac{1376\,K}{T}\right] \tag{S4a}$$

$$\sigma_{\beta} = 1.8 \tag{S4b}$$

$$A_{\beta}(T) = 0.000399 - \left(\frac{0.01436\,K}{T}\right) \tag{S4c}$$

These equations provided a good description of the experimental data below T_g and have been assumed to remain valid at higher temperatures.

The total dielectric loss relaxation of each pure component can be written as:

$$\varepsilon''(\omega) = \varepsilon''_{\beta}(\omega) + \varepsilon''_{\alpha}(\omega) \qquad \text{(S5)}$$

The HN parameters $\Delta\varepsilon$, α, γ of the SBR α-relaxation were determined by fitting the data in the temperature interval from 230K to 280 K assuming α and γ as temperature independent and Δε proportional to reciprocal temperature, $\Delta\varepsilon(T) = \Delta\varepsilon(T_g)\frac{T}{T_g}$. The parameters for PS were also determined with equivalent assumptions by fitting the data in the temperature interval from 290 K to 340 K. In these calculations we have used $\varepsilon_\infty = 2.35$ for SBR and $\varepsilon_\infty = 2.70$ for PS. Figure S1 shows that in this way a quite good description of the experimental data is obtained using a few parameters (see Table S1). The low-frequency increase of the results is due to conductivity effects, not taken into account in this analysis. The characteristic time at each temperature can be defined as the inverse of the angular frequency at the dielectric loss maximum of the α-relaxation process, which was calculated from the fitting parameters as: [2]

$$\tau_{max} \equiv \omega^{-1}_{max} = \tau_{HN}\left[\sin\left(\frac{\alpha\,\gamma\,\pi}{2+2\,\gamma}\right)\right]^{1/\alpha} \cdot \left[\sin\left(\frac{\alpha\,\pi}{2+2\,\gamma}\right)\right]^{-1/\alpha} \qquad \text{(S6)}$$

Figure S2 shows the temperature dependence of the τ_{max} for SBR and PS. The lines in the figure correspond to the data description by means of Vogel-Fulcher-Tammann (VFT) equation: [3-5]

$$\tau_{max}(T) = \tau_\infty \exp\left(\frac{DT_0}{T-T_0}\right) \qquad \text{(S7)}$$

Table S1 includes the values obtained for the fragility parameter, D, and the Vogel temperature T_0. In the fits we kept constant the prefactor value τ_∞=10^{-13}s in the VFT equation.

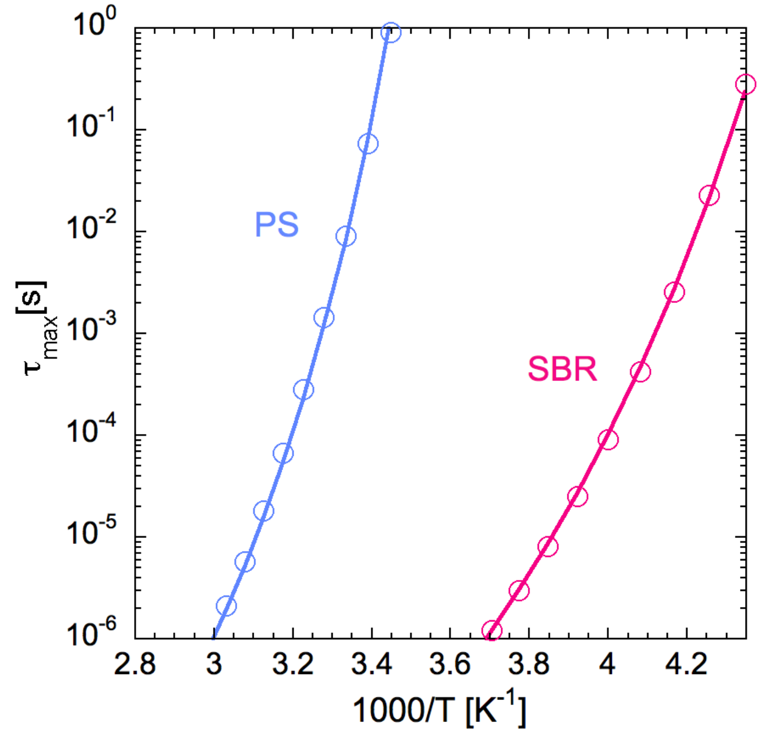

Figure S2. Temperature dependence of the characteristic times defined from the inverse of the frequencies of the dielectric loss maxima for the α-relaxation process of the samples investigated. The lines stand for the fits by means of the VFT equation.

Table S1: Parameters involved in the description of the dielectric α-relaxation of the pure components. The dielectric strength and the Havriliak-Negami parameters were obtained fitting the curves well centered in the experimental frequency window. In the VFT equation describing $\tau_{max}(T)$, τ_∞=10^{-13}s was fixed.

sample	α	Γ	$\Delta\varepsilon\ T/T_g$	D	$T_0 (K)$
SBR	0.54	0.7	0.096	8.6	176.7
PS	0.63	0.54	0.074	6.3	239.8

(ii) The dielectric α-relaxation of SBR/PS blends

For the different blends, Figure S3 shows the dielectric loss tangent as a function of frequency at 260 K (a) and at temperatures where the α-relaxation peak is well centered in the explored frequency window (b). Results are compared with those of the neat polymers in the same figure. From the shape and the position of the peaks, it is clear that the α-relaxation is strongly affected by blending. As we add PS to SBR in the blend, a broader and slower α-relaxation is observed compared to that of pure SBR. So, blending affects the α-relaxation by producing a broadening of the relaxation, evidencing the dynamic heterogeneity. However, the β-relaxation does not seem to be significantly affected by blend composition, beyond the relaxation intensity that is in good approximation proportional to the corresponding weight fraction.

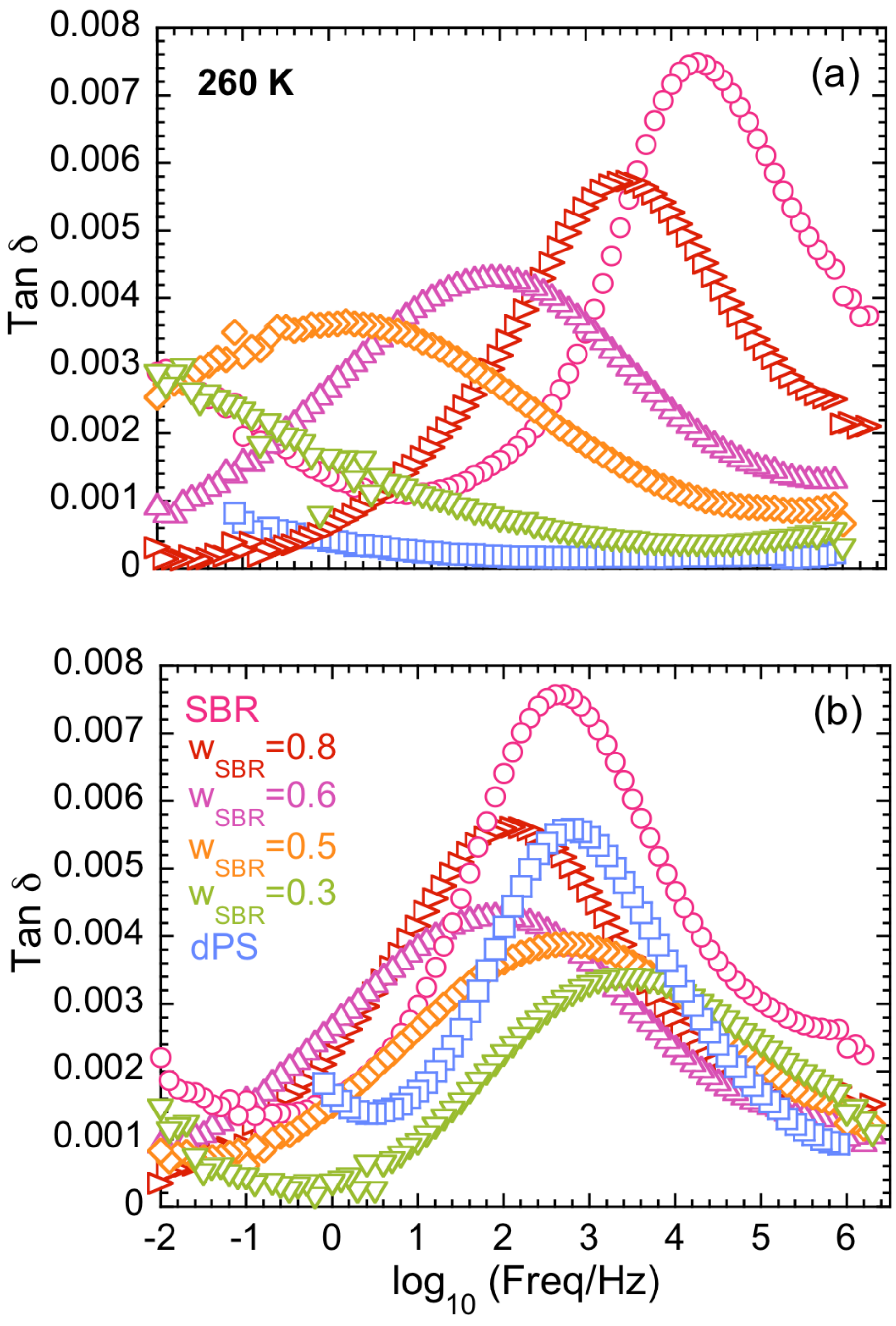

Figure S3. a) Dielectric spectra at 260 K from the pure components and from blends. b) Comparison of dielectric spectra at representative temperatures from the pure components and from blends. The corresponding temperatures are 245 K for SBR, 310 K for PS, 250 K for w_{SBR}=0.8, 260 K for w_{SBR}=0.6, 275 K for w_{SBR}=0.5, and 295 K for w_{SBR}=0.3.

As described in the main manuscript, the model for the blends is based on thermally driven concentration fluctuations (TCF) and self-concentration (SC) concepts.

Using this approach most of the parameters needed are those above determined for the pure component, assumed to be unaffected by blending, and summarized in Table S1.

In a previous work [6] the self-concentration values for SBR and PS in the blend ($\varphi_{self}^{SBR} = \varphi_{self}^{PS} = 0.2$) were assumed to be valid over the whole range of concentration and temperature; the only parameter to be determined by comparing the model calculations with the experimental data was σ. In a latter work,[7] from the comparison of BDS σ-values with those deduced from SANS for TCF (see the main text), we deduced that the relevant length scale for the α-relaxation as monitored by BDS $2R_c$ was close to 15 -- 20Å. We note that those works were performed on similar not yet the same SBR/PS blends, being the SBR of different microstructure and molecular weight, and PS in Refs. [6] and [7] was protonated. Here we have assumed that the SANS experiments can provide the concentration-dependent values of σ, if we impose a constant value of $2R_c$. We thus look for the value of $2R_c$ among those used in Figure 6 of the main manuscript to calculate the Gaussian functions widths from the SANS results providing the best description of the BDS results considering for each composition three different temperatures. For this end, we started with a fitting of the mixtures at intermediate temperatures by allowing the three parameters, φ_{self}^{SBR}, φ_{self}^{PS}, and σ to vary freely. The resulting σ values were compared with those calculated from SANS to determined the most adequate value of $2R_c$.

The so-determined $2R_c$-value was 25Å which yields $\sigma_{0.8}$ =0.09, $\sigma_{0.6}$ =0.11, $\sigma_{0.5}$ =0.115, and $\sigma_{0.3}$ =0.10. Note that by this procedure the uncertainty of 2Rc is around 5 Å (see Figure 6 of the main manuscript) and the corresponding uncertainty of σ is about 15%. With these fixed σ-values, we have run out again the minimization on the 4 mixtures, selecting for each case the temperatures where the relaxation peak is well centered in the experimental frequency window (see Figure S4), allowing φ_{self}^{SBR} and φ_{self}^{PS} to change. From the resulting values we obtained the corresponding average self-concentration values $\varphi_{self}^{SBR} = 0.14 \pm 0.04$ and $\varphi_{self}^{PS} = 0.19 \pm 0.05$, which in the following will be taken as temperature and composition independent.

As can be seen in Figure S4, in this way the model presented here allows to describe the dielectric relaxation of the SBR/PS blends, over a broad range of temperatures and compositions, in a very satisfactory way with only three free parameters, φ_{self}^{SBR} and φ_{self}^{PS}, accounting for summarized -concentration effects, and $2R_c$, which establishes the relevant length scale for the α-relaxation in these blends.

It is noteworthy that in the system here investigated and under the conditions we have considered to characterize the segmental dynamics confinement effects due to slow dynamics of the higher-T_g component[8] can be ruled out. In the present case the dynamic asymmetry of our systems (difference between the T_gs of the neat components) is moderate as compared to situations where confinement effects were of relevance[8] for the blends richer in the slower component.

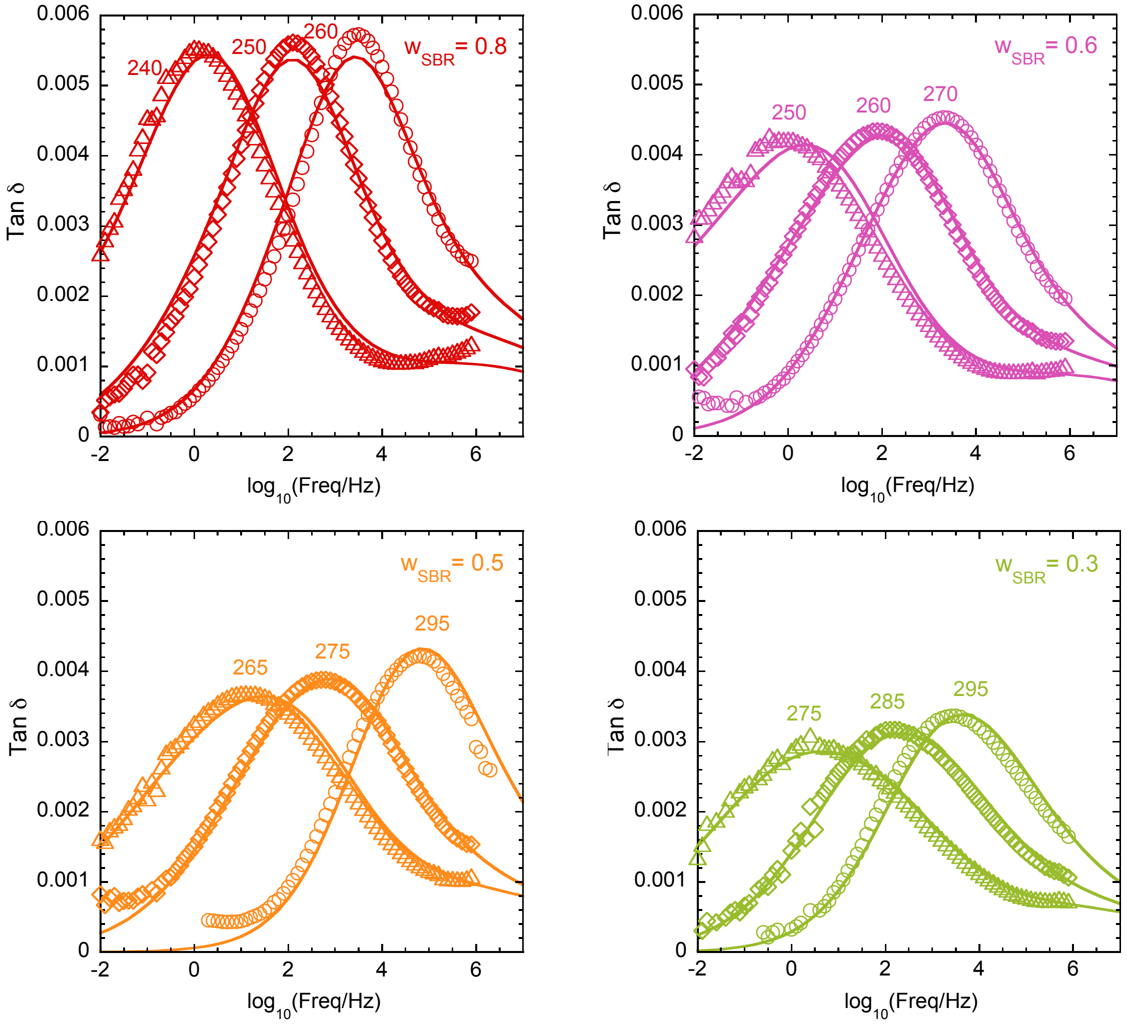

Figure S4. Dielectric loss tangent as a function of frequency for SBR/PS blends at three different temperatures where the main loss peak is centered. The corresponding composition and temperatures are also indicated. Solid lines correspond to the model description of the experimental data.

(iii) Derivative of the calorimetric traces of SBR/PS blends

The good agreement between the DSC traces and model is also corroborated when the temperature derivative of the DSC data and model curves are compared (see Figure S5). In this representation, the glass transition processes appear as peaks and the extension of the transition range is more clearly quantified; thereby, the good quality of the DSC data description is emphasized, both in the peak position and in the breadth of the glass transition range.

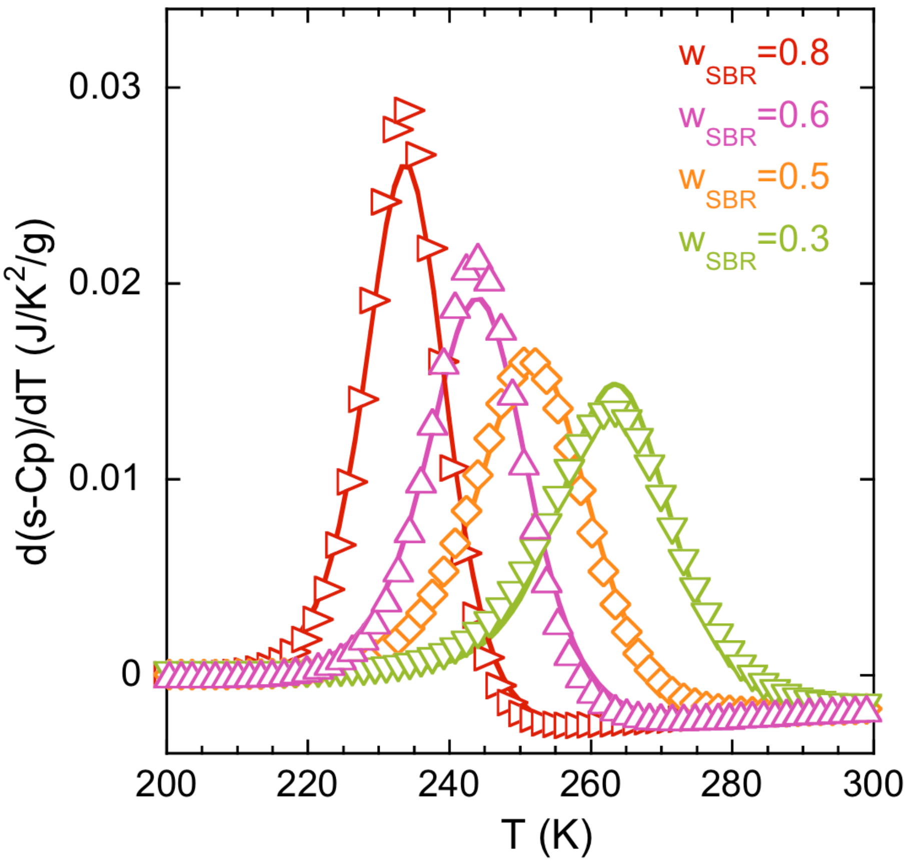

Figure S5. Comparison of the temperature derivative of the DSC data from Figure 8 with the corresponding model curves.

(iv) Determination of the effective interaction parameter from SANS

The Flory interaction parameter between PS and SBR χ can be obtained from the composition-dependence of the zero wavevector limit of the scattering function S(0). For a binary blend of interacting polymer chains of species A and B with corresponding degrees of polymerization N_A and N_B, monomeric volumes v_A and v_B and average volume fractions ϕ_A and $\phi_B = 1 - \phi_A$, the Random Phase Approximation (RPA) predicts that[9]

$$\frac{1}{S(0)} = \frac{(\Delta\rho)^2}{I(0)} = \frac{1}{N_A\langle\phi_A\rangle v_A} + \frac{1}{N_B(1-\langle\phi_A\rangle)v_B} - \frac{2\chi}{v_0} \qquad \text{(S8)}$$

Here v_o is the molar volume of a reference unit cell $v_o = (v_A v_B)^{1/2}$.The fits of the SANS results yield $I(0)=I_{OZ}(0)$. Using the values calculated for polymerization degrees, monomeric volumes and scattering length densities of the blend components (see main text), Eq. (S8) was fit to the experimentally obtained values of *S(0)* at different temperatures, as can be seen in Figure S6. We also considered additional SANS results obtained for a sample with higher concentration of PS (w_{SBR}=0.15), to increase the concentration range and reduce the uncertainties in the determination of the χ-parameter. Note that for this and the w_{SBR}=0.30 sample, the results at the lowest temperature (265K) were discarded, since it is very close to the average glass-transition of these blends and equilibration was probably not achieved. The χ-values obtained from the fits are represented in Figure S7. In the whole temperature range investigated the effective χ parameter presents positive values, indicative of repulsive interactions between the components. Its temperature dependence can be described by the law $\chi = -0.124 + 46.05K/T$. In Figure S7 the

values reported for the blend system investigated in Ref. [7] are also included for comparison. They follow the law $\chi = -0.0747 + 41.45K/T$.

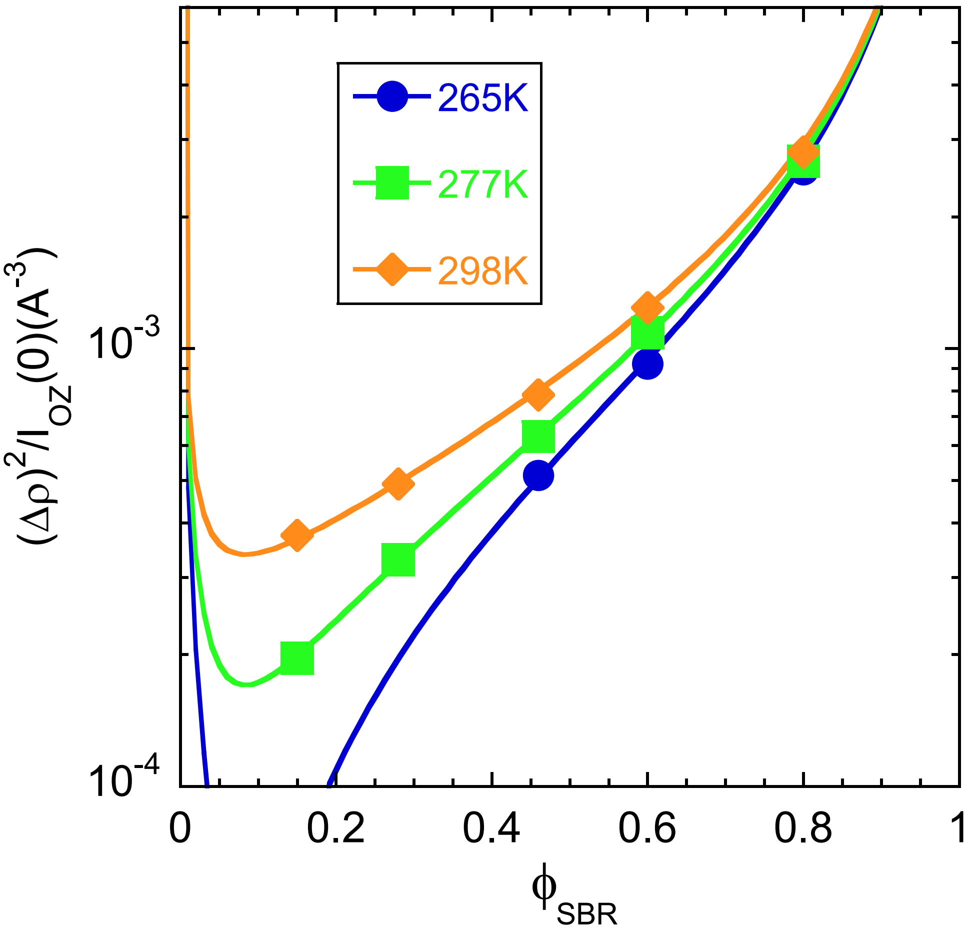

Figure S6. Concentration dependence of the inverse of the OZ amplitude for the three temperatures investigated. Lines are fits of Eq. (S8).

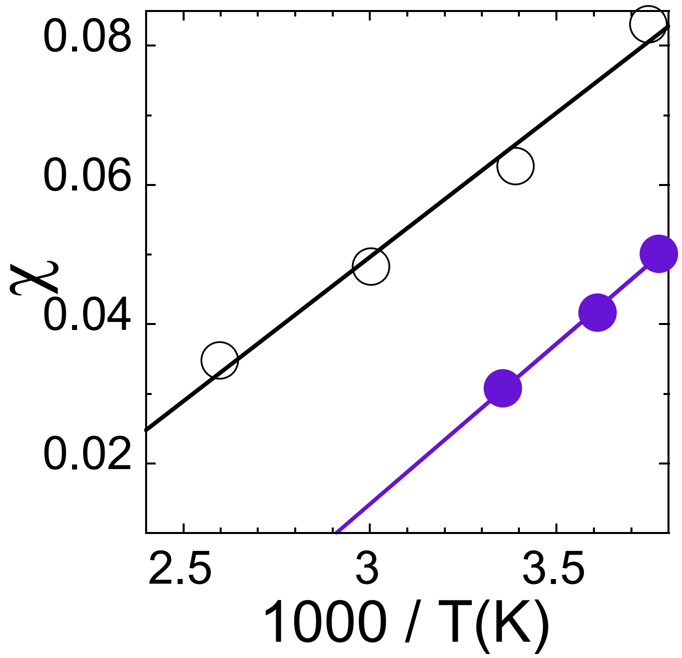

Figure S7. Inverse temperature dependence of the χ parameter obtained in this work (filled circles) and in Ref. [5] (empty circles). Lines are fits by the laws $\chi = -0.124 + 46.05K/T$ and $\chi = -0.0747 + 41.45K/T$, respectively.

(v) Temperature dependence of the width of concentration fluctuations

Figures S8 and S9 show the width of the Gaussian distributions of concentration fluctuations deduced from the SANS results for a spherical relevant volume of $2R_c$ diameter for different temperatures.

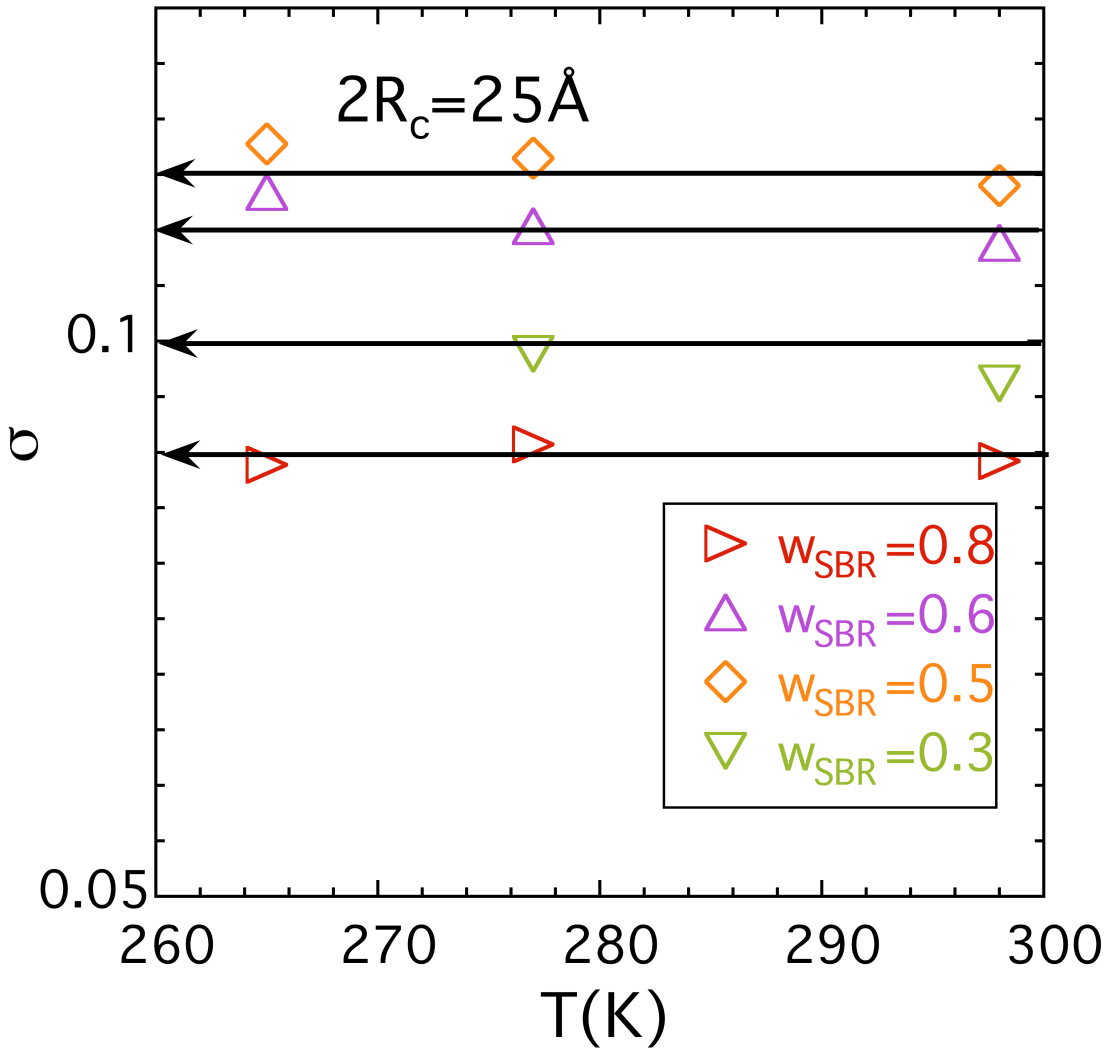

Figure S8. Temperature dependence of the width of the Gaussian distributions of concentration fluctuations deduced from the SANS results for a spherical relevant volume of 25Å diameter. Arrows mark the values that have been imposed in the application of the model.

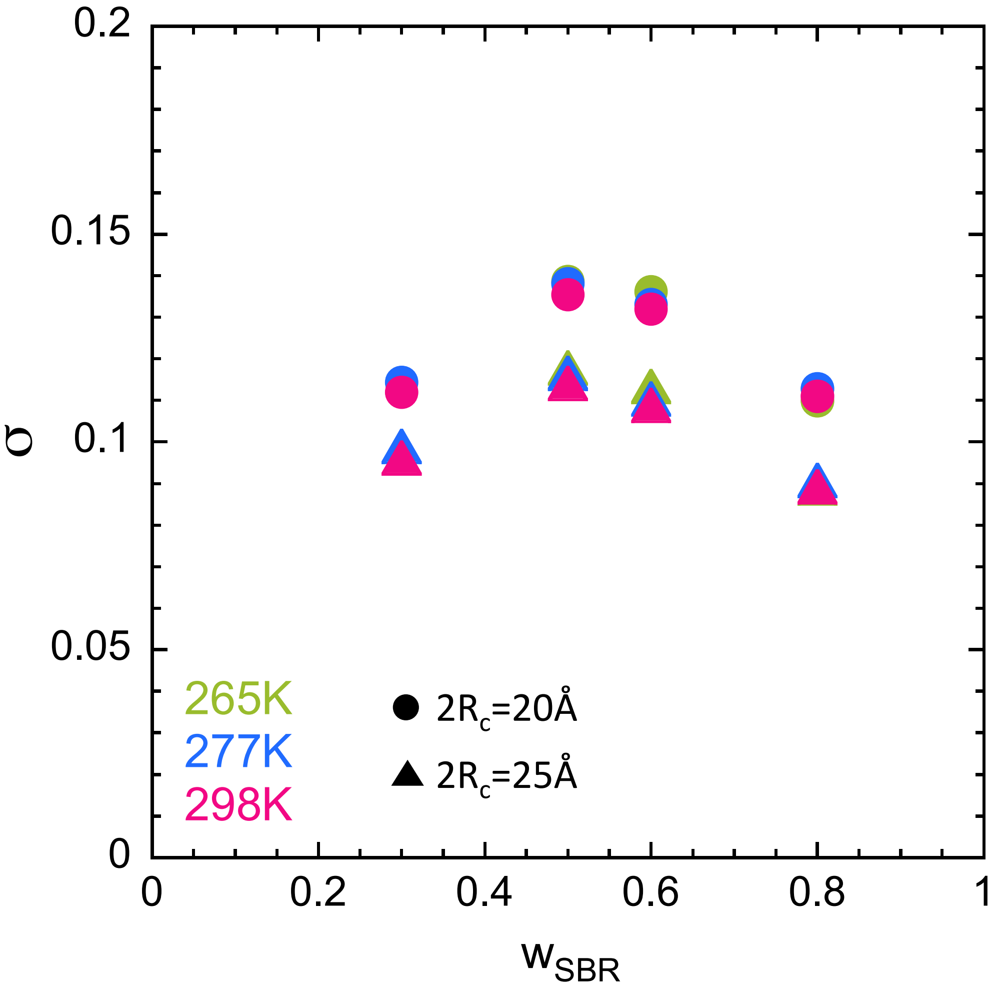

Figure S9. Comparison of the temperature dependence of the width of the Gaussian distributions of concentration fluctuations deduced from the SANS for two different values of the diameter. The observed variations are in all cases smaller than the typical uncertainty in the determination of σ.

REFERENCES

(1) Alvarez, F.; Alegría, A.; Colmenero, J. Interconnection between Frequency-Domain Havriliak-Negami and Time-Domain Kohlrausch-Williams-Watts Relaxation Functions. *Physical Review B* **1993**, *47*, 125–130.

(2) Kremer, A., Friedrich; Schönhals, *Ed. Broadband Dielectric Spectroscopy*; Springer: **2003**.

(3) Vogel, H. The Law of the Relation between the Viscosity of Liquids and the Temperature. *Physikalische Zeitschrift* **1921**, *22*, 645.

(4) Fulcher, G. S. Analysis of Recent Measurements of the Viscosity of Glasses. *Journal of the American Ceramic Society* **1925**, *8*, 339–355.

(5) Tammann, G.; Hesse, W. Die Abhängigkeit der Viscosität von der Temperatur bei unterkühlten Flüssigkeiten. *Zeitschrift für anorganische und allgemeine Chemie* **1926**, *156*, 245–257.

(6) Gambino, T.; Alegría, A.; Arbe, A.; Colmenero, J.; Malicki, N.; Dronet, S. Modeling the High Frequency Mechanical Relaxation of Simplified Industrial Polymer Mixtures Using Dielectric Relaxation Results. *Polymer* **2020**, *187*, 122051.

(7) Gambino, T.; Shafqat, N.; Alegria, A.; Malicki, N.; Dronet, S.; Radulescu, A.; Nemkovski, K.; Arbe, A.; Colmenero, J. Concentration Fluctuations and Nanosegregation in a Simplified Industrial Blend with Large Dynamic Asymmetry. *Macromolecules* **2020**, *53*, 7150–7160.

(8) Colmenero, J.; Arbe, A. Segmental Dynamics in Miscible Polymer Blends: Recent Results and Open Questions. *Soft Matter* **2007**, *3*, 1474–1485.

(9) Rubistein, M.; Colby, R. H. *Polymer Physics*, Oxford University Press, Oxford, **2003.**
